# Short-term effects of COVID-19 on the risk of traumatic fractures in China cities

**DOI:** 10.1038/s41598-022-10531-2

**Published:** 2022-04-20

**Authors:** Hongzhi Lv, Xiaolin Zhang, Juan Wang, Zhiyong Hou, Haicheng Wang, Chao Li, Wenjuan Wang, Wei Chen, Yingze Zhang

**Affiliations:** 1 Department of Orthopaedic Surgery, The Third Hospital of Hebei Medical University, No. 139 Ziqiang Road, Shijiazhuang, 050051 China; 2 Department of Epidemiology and Statistics, Hebei Medical University, Shijiazhuang, 050017 China; 3 NHC Key Laboratory of Intelligent Orthopaedic Equipment, The Third Hospital of Hebei Medical University, No. 139 Ziqiang Road, Shijiazhuang, 050051 China

**Keywords:** Diseases, Medical research

## Abstract

This study aimed to investigate the association between COVID-19 and fracture risk and provide a targeted reference for the world through China’s experience. A nationally representative sample of COVID-19 prevalence areas selected using stratified random sampling was retrospectively analyzed. Age, sex, fracture site, mechanism of injury, and concurrent fractures of traumatic fracture patients in selected hospitals were collected from 10 January to 10 July 2020. The epidemiologic characteristics of traumatic fractures and the association between COVID-19 and fracture risk were explored using descriptive epidemiological methods and a distributed lag nonlinear model. A total of 67,249 patients (52.3% males, 49.4 ± 19.4 years old) with 68,989 fractures were included. The highest proportion of fractures were in the tibia and fibula (14.9%), followed by the femur (13.6%) and ulna and radius (12.5%). Low-energy fractures accounted for 23.3%. With the increase in newly confirmed COVID-19 cases, fracture risk decreased for children, young and middle-aged adults, elderly men, high-energy fractures, and residents in regions with < 1000 cumulative confirmed COVID-19 cases. Fracture risk decreased sharply in all residents except elderly women, for low-energy fractures, and in regions with > 1000 cumulative confirmed COVID-19 cases when newly confirmed COVID-19 cases increased in China. Primary (home) prevention measures are emphasized to prevent traumatic fractures.

## Introduction

Coronavirus disease (COVID-19) has swept the world and has officially been declared a global pandemic^1–5^. By 26 November 2020, the outbreak of COVID-19 had generated more than 60,864,066 confirmed cases in 208 countries, including 1,429,812 deaths (https://covid19.who.int/). Many countries have adopted strict preventive control measures to restrict people’s movement, including the wearing of masks, self-isolation at home, traffic control, and community blockade. Although China has suffered from the COVID-19 outbreak since 20 January 2020, the Chinese government has taken strict prevention and movement-restricting measures that have proved to be very effective in controlling and wiping out the pandemic. Since March 18 2021, there have been no new confirmed native cases in China; all new confirmed cases were imported (https://covid19.who.int/region/wpro/country/cn).

Traumatic injury is one of the leading causes of death and disability worldwide. Injuries also place a huge burden on China; there, they are the fifth most common cause of death and result in more deaths than do diabetes and infectious diseases^6^. Injury-related fractures represent a primary drain on medical resources^7,8^. The spread of COVID-19 is associated with the occurrence of traumatic fractures due to changes in individuals’ lifestyles and psychological states. Several studies have reported that preventive measures such as self-isolation at home, traffic control, and strict limitation of individuals’ access to the community has decreased the risk of traumatic fractures^9,10^. To date, however, the effect of COVID-19 infection on traumatic fractures has not been investigated extensively.

It is important to investigate the epidemiology of patients with COVID-19 and identify the risk factors for traumatic fractures. The purpose of this study was to evaluate the relationship between traumatic fractures and the spread of COVID-19. We also evaluated whether this relationship changed according to age, sex, site, mechanism of the injury, or the area of the epidemic in which the traumatic fractures occurred.

## Patients and methods

### Data sources

#### Sampling methods

**Figure 1:**
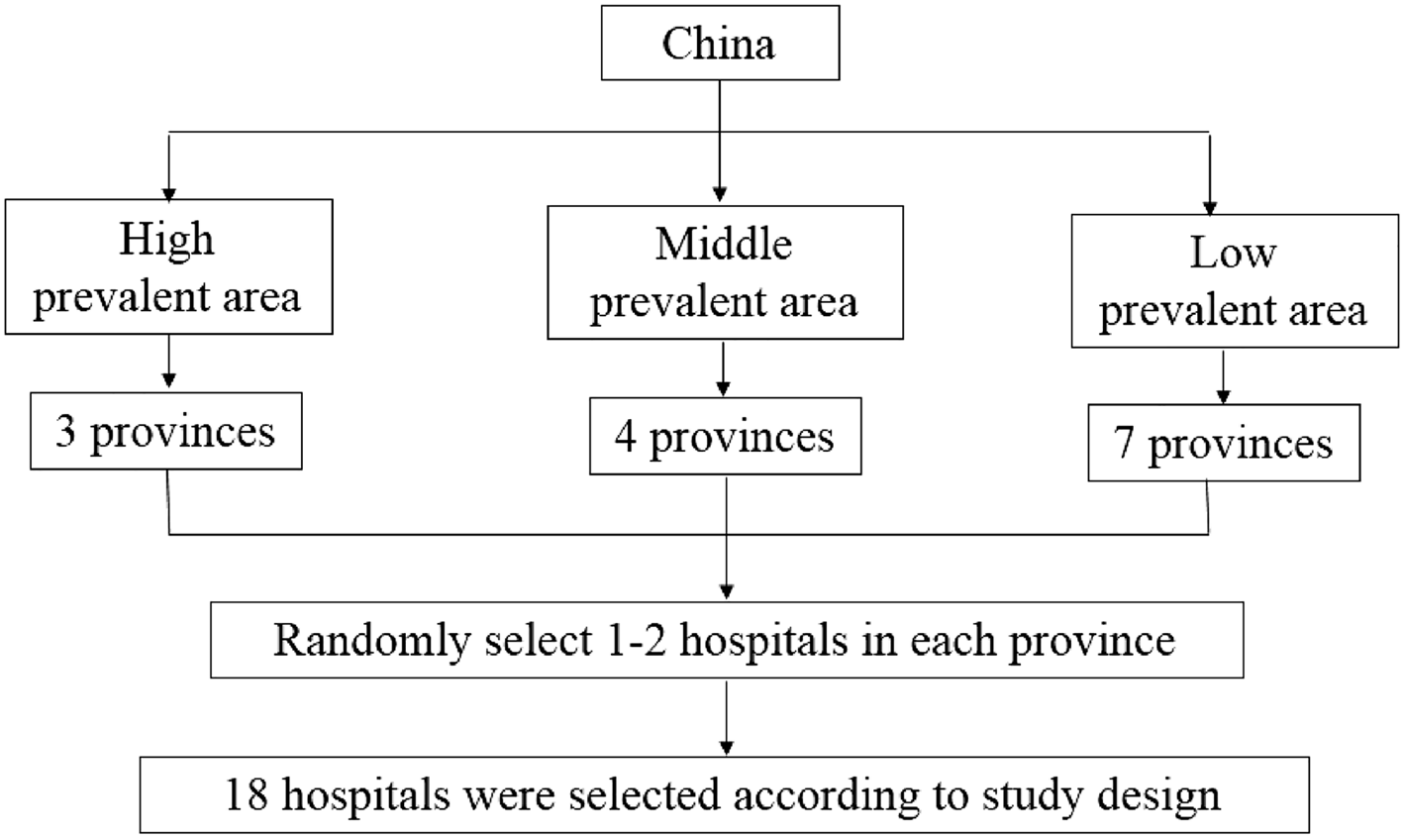
Flow chart of the survey sampling process.

This was a retrospective survey. During the main sampling phase, 31 provinces (municipalities or autonomous regions) in mainland China were categorized according to which of three types of regions they were located in regions with > 1000 cumulative confirmed cases, regions with 500–1000 cumulative confirmed cases, and regions with < 500 cumulative confirmed cases according to the available data on the cumulative number of confirmed cases in each province in China as of 10 July 2020 (http://www.nhc.gov.cn/xcs/yqtb/list_gzbd.shtml). Fourteen provinces and municipalities were calculated with the optimum allocation stratified random sampling survey (three in regions with > 1000 cumulative confirmed cases, four in regions with 500–1000 cumulative confirmed cases, and seven in regions with < 500 cumulative confirmed cases, as shown in the flow chart in Fig. 1). Within each targeted province, one or two hospitals were randomly selected. In cases in which the hospital manager refused to allow the hospital to participate, an alternative hospital was randomly selected from the list using a modified version of the Kish method^11^. Finally, 19 hospitals were selected; they included the Third Hospital of Hebei Medical University, Beihai People’s Hospital, Jingxing County Hospital, People’s Hospital of Peking University, Beijing Jishuitan Hospital, The First Affiliated Hospital of Dalian Medical University, The First Affiliated Hospital of Fujian Medical University, Fuzhou No. 2 Hospital, Henan Provincial People’s Hospital, China-Japan Union Hospital, Hospital of Jilin University, Jiangsu Provincial People’s Hospital, Nanfang Hospital Affiliated with Southern Medical University, Affiliated Hospital of Nantong University, Affiliated Hospital of Qingdao University, Shanghai No. 6 People’s Hospital, Tianjin Hospital, Wuhan Union Hospital, and The First Affiliated Hospital of Xinjiang Medical University. Of these, 18 are tertiary referral hospitals and one is a secondary referral hospital.

This study was approved by the Institutional Review Board of the Third Hospital of Hebei Medical University and was conducted in compliance with the Helsinki Declaration; consent was waived due to its retrospective nature.

#### Inclusion and exclusion criteria

The inclusion criteria were as follows: (1) definite diagnosis of new-onset fracture; and (2) the fracture was sustained between 10 January and 10 July 2020. The exclusion criteria were (1) pathologic (metastatic) fracture of a bone weakened by tumor or disease, an event that often occurs due to little trauma or even without obvious trauma and is most commonly caused by primary or metastatic bone tumors, osteoporosis, endocrine disorders, or developmental disorders of bone and cartilage^12–14^; (2) secondary fracture arising from various causes, including poor union, nonunion or readmission, and periprosthetic fracture.

### Data collection and groups

All cases of fractures treated in the selected hospitals from 10 January to 10 July 2020 were collected through the Picture Archiving and Communication System (PACS) and the case report checking systems. The collected data of interest included demographic information (age and sex), fracture site, mechanism of injury, and concurrent fractures. All medical charts and radiographs of fracture patients in each participating hospital were evaluated by two local orthopedic surgeons, and any inconsistencies were addressed by discussion. The patients were divided into three groups based on age: children (≤ 14 years), young and middle-aged adults (15–64 years), and older patients (≥ 65 years). Subjects were also stratified into the following age groups: 0–4 years, 5–14 years, followed by an age group of 10 years, and ≥ 65 years.

The fracture sites were recorded as proximal, shaft, or distal for each limb long-bone (humerus, ulna and radius, femur, and tibia and fibula); as pelvic or acetabular; scapular; clavicular; patellar; spinal; hand and wrist; foot and ankle; and other, including sternum, rib, and head fractures. Patients who met the following three criteria were considered to have sustained osteoporotic fracture: (1) the fracture occurred either in the hip, thoracic or lumbar vertebrae, the distal radius, or the proximal humerus; (2) were ≥ 65 years old, and (3) had a low-energy injury^15–19^.

The mechanism of injury included low-energy and high-energy fractures. A low-energy fracture was defined as a fracture caused by a fall from standing or from a low height (< 1 m). A high-energy fracture was defined as a fracture caused by a traffic accident, blunt injury, injury with a sharp instrument, fall from a high height, explosive crush injury, and others.

#### COVID-19 cases

The daily average numbers of COVID-19 cases were obtained from the National Health Commission of the People’s Republic of China platform. The system performs the quality assurance and quality control tasks of the Chinese government. The monitoring station built by the National Health Commission of the People’s Republic of China provides COVID-19 data to the system daily to continuously monitor COVID-19 prevalence throughout China. From January 10 to July 10, 2020, which was the most serious period of the COVID-19 epidemic, there were 183 newly diagnosed cases. The principles followed in the management of traumatic fracture during the COVID-19 epidemic are summarized in Supplementary File 1.

### Statistical analyses

Statistical analyses were conducted using SPSS 23.0 statistical software (IBM, Armonk, New York, USA). The Kolmogorov–Smirnov test was used to test whether the data conformed to a normal distribution. The *t* test of two independent samples was used to compare the ages of different sexes in accordance with the normal distribution. ANOVA was used to compare the ages of patients in different areas of the epidemic.

Modeling was performed using R 2.15.0 (R Foundation for Statistical Computing, Vienna, Austria). We studied the impact of COVID-19 on the number of fracture cases in a distributed lag model. In this model, the dependent variable is the number of newly confirmed traumatic fractures per day, and the independent variable is the number of newly confirmed COVID-19 cases per day for each day within a 10-day window. The model is constructed by establishing a lagging cross-basis matrix of the numbers of newly diagnosed COVID-19 cases and adding the natural cubic spline function of time α and the week variable and holidays as covariates to control the confounding of trend and week effect.

The final distributed lag model is as follows:

Yt ~ quasi-Poisson (fracture cases).

$$ {\text{Log }}\left( {{\text{fracture}}\;\;{\text{ cases}}} \right) = \alpha + W_{X}^{n} \eta + {\text{ns }}\left( {{\text{time}},{ 7}*{\text{year}}} \right) + \beta_{{3}} *{\text{DoW}} + \beta_{{4}} *{\text{Holidays}}. $$

“${W}_{X}^{n}$ ŋ” represents the cross-basis function. “DoW” represents the day of the week. “Holidays” represents holidays and vacations.

### Ethical approval

This study was approved by the Institutional Review Board of the Third Hospital of Hebei Medical University and registered on the Chinese Clinical Trial Registry (Registration number: ChiCTR-EPR-15005878).

## Results

### Descriptive statistics of the data

Within the study timeframe, there were a total of 67,249 patients with 68,989 fractures; of these patients, 65,715 (95.3%) had single fractures, 1377 (20%) had two concurrent fractures, 125 (2%) had three concurrent fractures, 18 (0.2%) had four concurrent fractures; 11 (0.1%) had five concurrent fractures, and three (0.4%) had six concurrent fractures. In the Wuhan Union Hospital, two fracture patients were confirmed to have COVID-19 on March 1, and one patient was confirmed to have COVID-19 on February 1; these patients included one with femoral neck fracture, one with calcaneal fracture, and one with lumbar fracture.

**Figure 2:**
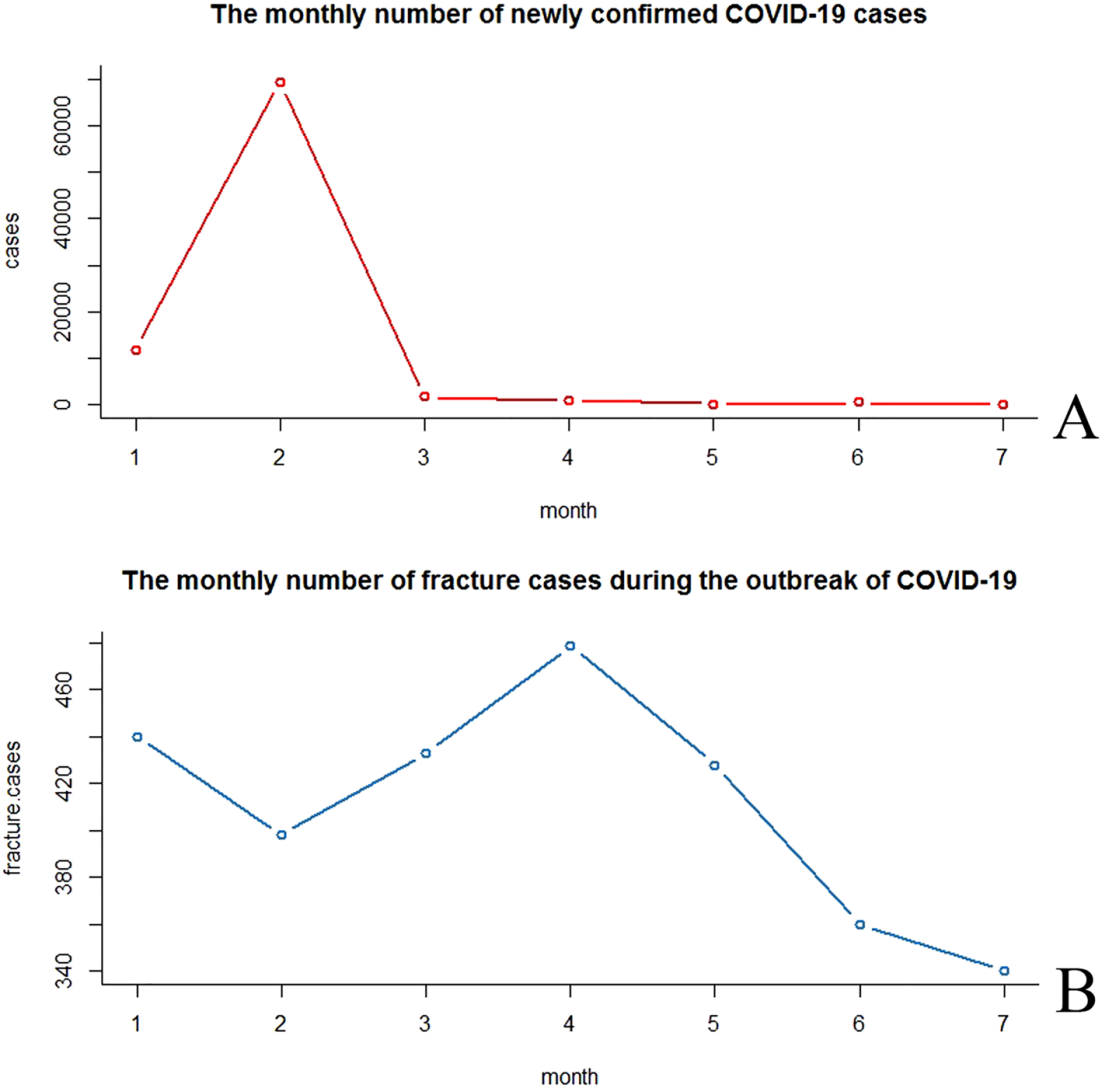
Monthly numbers of newly confirmed COVID-19 cases and fracture cases.

A total of 84,622 newly confirmed COVID-19 cases were recorded from January 10 to July 10, 2020. The number of newly confirmed COVID-19 cases was highest in February (69,400) (Fig. 2a), while the number of fracture cases was highest in April (479) (Fig. 2b).

### Age- and gender-specific characteristics

**Figure 3:**
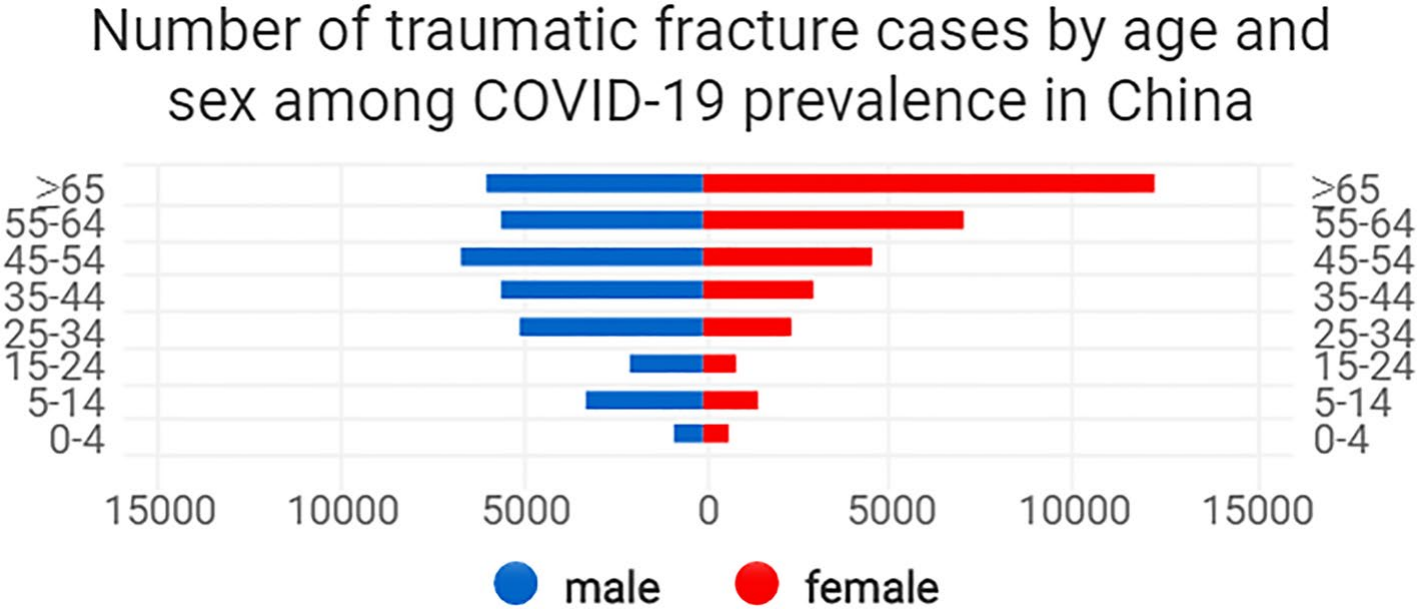
Number of traumatic fracture cases by age and sex that occurred during the period of COVID-19 prevalence in China.

There were 35,176 (52.3%) male and 32,073 (47.7%) female patients, with an average age of 49.4 ± 19.4 years (range, 1–108). The mean age of the males was 43.9 ± 20.6 years, significantly lower than that of females (55.4 ± 21.0 years; *t* = − 71.434, *P* < 0.01). This study collected data on 6,196 (9.2%) children, 42,800 (63.6%) young and middle-aged adults, and 18,235 (27.1%) elderly patients. The ratios of males to females in these age groups were 1.9, 1.4, and 0.5, respectively. It can be seen from the age-sex pyramid of patient composition that the majority of patients were females over 65 years of age (Fig. 3).

### Regional characteristics

**Figure 4:**
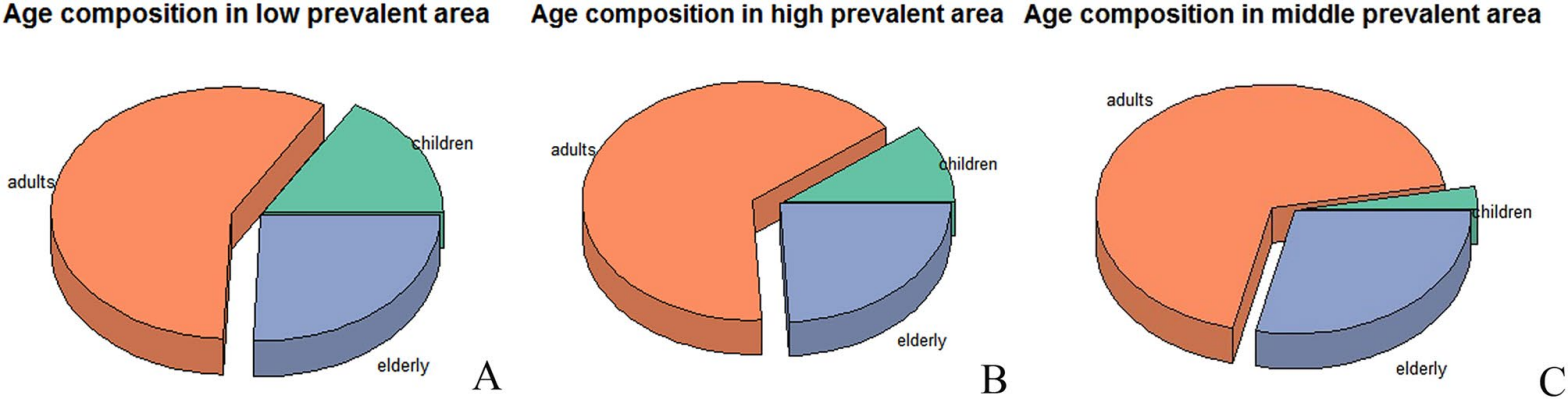
Age composition of individuals with traumatic fracture in regions of China classified according to COVID-19 prevalence.

The mean age of fracture patients in regions with 500–1000 cumulative confirmed cases was 52.1 ± 18.9 years, significantly older than that in regions with < 500 (47.0 ± 22.1) and > 1000 (46.4 ± 23.9) confirmed cases (*F* = 580.839, *P* < 0.01). There were more males than females in regions with > 1000 (1025/760) and < 500 (16,528/13,553) cumulative confirmed cases and approximately the same number of males and females in regions with 500–1000 cumulative confirmed cases (17,623/17,760). Adults accounted for the highest proportion of cases in all regions, but the proportion of elderly individuals in regions with > 1000 cumulative confirmed cases (Fig. 4B) was lower than that in regions with < 500 (Fig. 4A) or 500–1000 cases (Fig. 4C; *χ*^2^ = 3493.108, *P* < 0.01).

### Fracture site and injury mechanism characteristics

**Figure 5:**
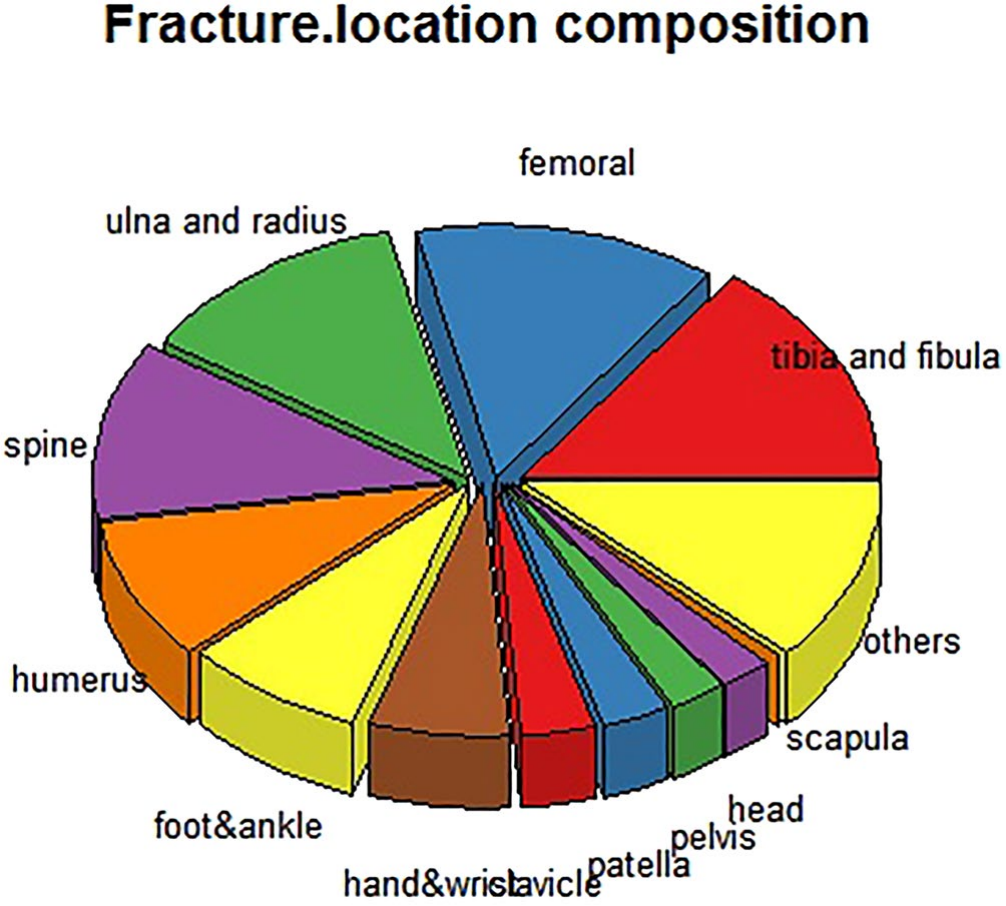
Locations of fractures that occurred during the period of COVID-19 prevalence in China.

Among 68,989 fractures, there were 10,275 tibia and fibula fractures, accounting for 14.9%, followed by femoral (9405, 13.6%), ulnar and radial (8598, 12.5%), other (8159, 11.8%), spinal (7923, 11.5%); humeral (6362, 9.2%), foot and ankle (5591, 8.1%), hand and wrist (4409, 6.4%), clavicular (2339, 3.4%), patellar (2076, 3.0%), pelvic and acetabular (1801, 2.6%), head (1579, 2.3%), and scapular (472, 0.7%) fractures (Fig. 5). Single fractures accounted for 97.7% (65,712) of the cases, and multiple fractures accounted for 2.2% (1537). There were 46,716 (67.7%) limb fractures and 22,273 (32.3%) trunk fractures.

**Figure 6:**
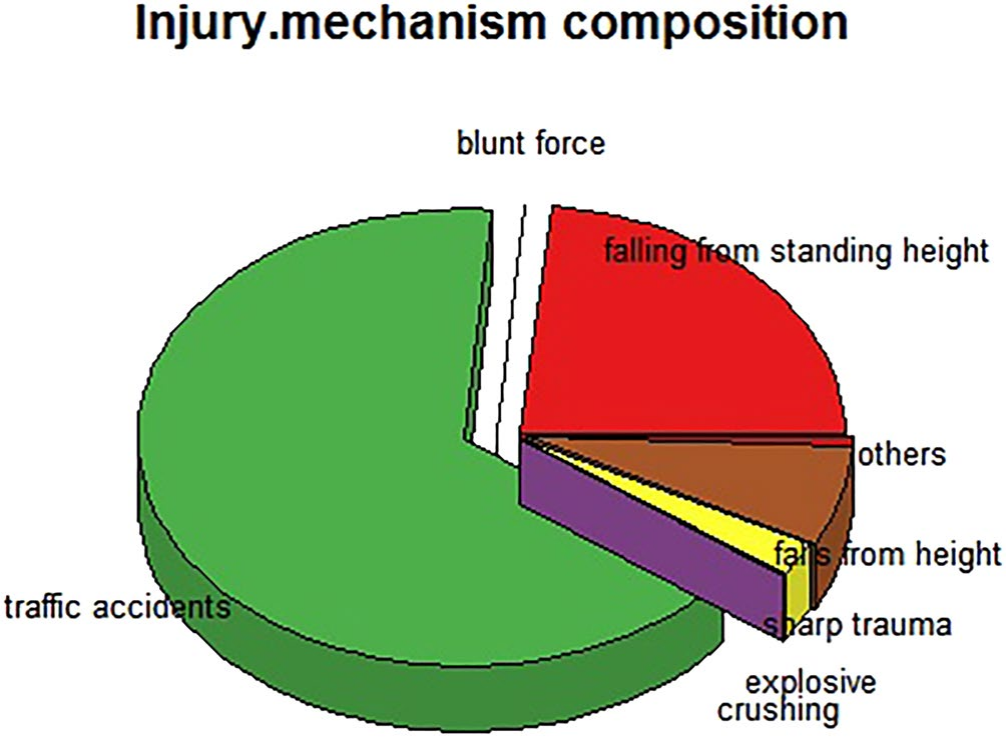
Injury mechanism composition of fractures that occurred during the period of COVID-19 prevalence in China.

Among 67,249 patients, the proportion with fractures caused by falling from a standing height was 23.3% (15,688/67,249). Other causes included traffic accidents (44,302, 65.9%), blunt force trauma (231, 0.3%), crushing injury (133, 0.2%), other (428, 0.6%), explosive injury (115, 0.2%), trauma caused by a sharp object (1571, 2.3%), and falls from heights (4781, 7.1%) (Fig. 6).

### Correlations among variables

#### All fracture cases

**Figure 7:**
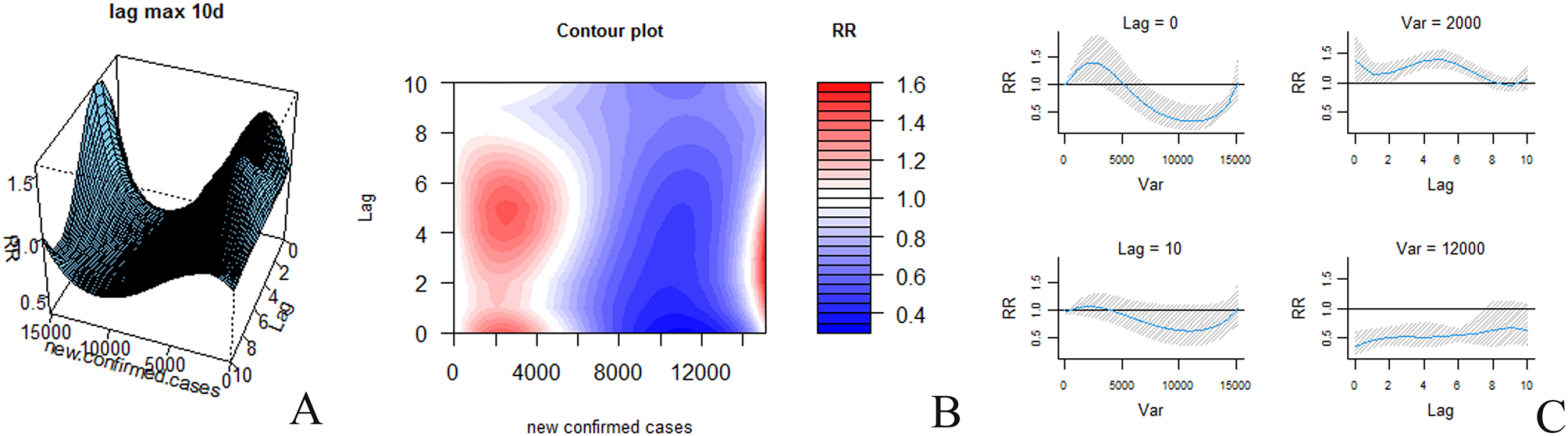
Relationship between the number of newly confirmed COVID-19 cases and fracture risk in China. (**A**) Shows all fractures in three-dimensional graphs, (**B**) shows two-dimensional contour plots, and (**C**) shows two-dimensional graphs for different numbers of lag days and newly confirmed COVID-19 cases.

Associations between COVID-19 incidence and fracture risk are presented as three-dimensional graphs and two-dimensional contour plots in Fig. 7. The daily number of newly confirmed cases ranged from 0 to 15,152, and the lag interval was 0–10 days. Figure 7A shows that as the number of newly confirmed COVID-19 cases increased, the fracture risk first increased and then decreased. As shown in Supplementary Table 1, on Days 0–7 of the lag interval, during which time the number of newly confirmed COVID-19 cases increased, the fracture risk first increased and then decreased (*P* < 0.05). Between the eighth and the tenth days of the lag interval, the fracture risk did not change with the increase in the number of newly confirmed COVID-19 cases (*P* > 0.05). The contour plot shows a profile of RR of newly confirmed cases with different concentrations and lag days, presenting the relationship among the three factors in a more intuitive way. The “slice” diagram of the RR shows that as the number of newly confirmed COVID-19 cases increased, the risk of fracture gradually decreased (Fig. 7B). Figure 7C shows the two-dimensional exposure lag response correlation. On day zero of the lag interval, with the increase in the number of newly confirmed COVID-19 cases, the fracture risk first increased and then decreased. On the tenth day of the lag interval, the fracture risk did not change with the increase in the number of newly confirmed COVID-19 cases. When the number of newly confirmed cases was 2,000, the fracture risk at 0–10 days of lag was higher, reaching a peak on the fifth day (RR 1.40, 95% CI 1.00–1.26). When the number of new confirmed cases was 12,000, the fracture risk at 0–10 days of lag was lower (RR < 1).

#### Gender and age

The fracture risk for males and females decreased with the increase in newly confirmed COVID-19 cases (Supplementary Fig. 1A,B). Supplementary Fig. 1C–G shows that with the increase in newly confirmed COVID-19 cases, the fracture risk decreased for children, young and middle-aged males and females, and elderly men but showed little change for elderly women.

#### Prevalence area

Supplementary Fig. 1H–J shows the impact of COVID-19 on the number of fracture cases in different regions. With the increase in the number of newly confirmed COVID-19 cases, fracture risk at 0–7 days of lag decreased in the regions with 500–1000 and < 500 cumulative confirmed cases. With the increase in newly confirmed COVID-19 cases, the fracture risk did not change in the regions with > 1000 cumulative confirmed cases.

#### Fracture location and mechanism of injury

Supplementary Fig. 1K–L shows that with the increase in the number of newly confirmed COVID-19 cases, limb and trunk fracture risk decreased. Supplementary Fig. 1M–N shows that with the increase in the number of newly confirmed COVID-19 cases, single fracture risk also decreased, while the risk of multiple fractures showed no change. Supplementary Fig. 1O–P shows that with the increase in newly confirmed COVID-19 cases, the risk of high-energy fracture decreased significantly, whereas no decrease in low-energy fracture risk was observed.

## Discussion

The present study found direct evidence of an effect of COVID-19 on fracture cases in China. This study compared the data on fractures treated at 18 hospitals in China within half a year of the COVID-19 outbreak and showed that the relationship between COVID-19 and fracture cases is described by an S-shaped curve; with the increase in the number of newly confirmed COVID-19 cases, the fracture risk for males, females, children, young men, young women, and elderly men decreased in regions with < 1000 cumulative confirmed cases and the risk of limb and trunk fracture was significantly reduced. These influences appeared after 0–7 days of lag and were no longer apparent after the eighth day.

The risk of fracture could be influenced by the COVID-19 pandemic. During the epidemic period, the government implemented a series of measures that limited travel and reduced population flow^9,10,20^. In many Chinese cities, supermarkets and shopping malls carried less than 5% of the patron volume that occurred during the nonepidemic period on January 23, 2020^9^. Between February 19 and the time at which the number of newly diagnosed patients was zero^10^, almost all residents except staff, doctors, and administrators engaged in prevention work were isolated in their own homes. Our findings suggest that with the increase in newly confirmed COVID-19 cases, the fracture risk for males, females, children, young men, young women, and elderly men decreased. This occurred because during the epidemic period, some industries were shut down and production was stopped, and the risk of fracture in young and middle-aged people was consequently reduced; the risk of fracture in children was also reduced after the suspension of school^20^. However, due to the reduction in exercise and the change in sedentary lifestyle during the epidemic period, elderly people who usually stay at home, especially elderly women with severe osteoporosis, were more likely to sustain low-energy fractures. The risk of fracture did not decrease as the epidemic situation intensified^9,10^. Therefore, in an epidemic period, we should consider implementing targeted fracture prevention and control measures at home, especially for elderly women. These measures can include correcting insufficient lighting and uneven floors in living rooms and washrooms, using proper walking aids, wearing antiskid shoes, and arranging furniture so as to avoid obstructions^21–23^. In the same way, for patients with hypertension, diabetes, and other diseases, we should ensure the timely and rational use of medication and reduce or eliminate the use of psychotropic drugs such as sleeping pills^24,25^.

In this study, with the increase in newly confirmed COVID-19 cases, fracture risk decreased in the regions with < 1000 cumulative confirmed cases at 0–7 days of lag. However, the fracture risk did not change in the regions with > 1000 cumulative confirmed cases. This might be due to the ceiling effect: provinces with > 1000 cumulative confirmed cases tended to adopt the most strict pandemic control measures, and this might have restricted personnel flow and decreased bone fracture risk to the lowest limit and thus not produced an observable effect.

In this study, with the increase in the epidemic situation, the number of single, limb, and trunk fractures markedly decreased, while low-energy fractures did not decrease and still accounted for 23.3% of all injury mechanisms. This is worthy of attention. The occurrence of many low-energy fractures will not only increase the consumption of scarce medical resources but can also lead to disability, reduce patients’ quality of life, and even cause death^26–28^. The need of fracture patients for long-term medical care will increase the chance that COVID-19 infection will occur in hospitals^29–31^. There is also increased evidence indicating that the incidence of COVID-19 in elderly individuals and its mortality rate in elderly patients are very high^32–34^. In addition, in the context of COVID-19, less physical labor, sedentary lifestyles, panic, and depressed mental state increase the risk of low-energy fractures due to falls and slips^35,36^. Therefore, attention should be given to the prevention of low-energy fractures during the COVID-19 epidemic. To avoid the occurrence of low-energy fractures, treatment of osteoporosis should be actively undertaken, and calcium supplements and active vitamin D3 should be used to promote the absorption of calcium^37–39^. We should simultaneously guide people on healthy home-based sports activities as well as on ways to self-regulate their emotional and psychological states.

This study has several limitations. First, it has a retrospective design, which inevitably leads to recall bias. However, the data were collected through the PACS and the case report examination systems. Because the main information collected includes demographic information (age and sex) and information on fracture site, injury mechanism, and other basic information, the recall bias of this study is very small. Second, the number of clinically diagnosed cases was included in the number of confirmed cases according to the novel coronavirus infection diagnosis and treatment plan (5th trial version) (http://www.gov.cn/zhengce/zhengceku/2020-02/05/content_5474791.htm) on February 12, 2020. This led to a sharp increase in the number of COVID-19 cases reported on that day, and this affected the overall flow of the model. Third, hospitals, rather than individuals, were randomly selected using the sampling method, as directly selecting individuals randomly in each administrative village or neighborhood community in China using this method would not have been practical.

## Supplementary Information

Supplementary Legends.

Supplementary Figure 1.

Supplementary Information.

Supplementary Table 1.

## Data Availability

The datasets used and analyzed during the current study are available from the corresponding author on reasonable request.
