# Supplementary material for: Short-term effects of COVID-19 on the risk of traumatic fractures in China cities
Source: Sci Rep. 2022 Apr 20;12:6528. doi: 10.1038/s41598-022-10531-2 (PMC9020760; doi:10.1038/s41598-022-10531-2)
Supplement: Supplementary file 1 — Supplementary Legends. [file 41598_2022_10531_MOESM1_ESM.docx]

**Supplementary Information**

**Supplementary File 1.** Principles followed in the management of traumatic fracture during the COVID-19 epidemic

**Supplementary Table 1.** Relationship between the number of newly confirmed COVID-19 cases and the number of traumatic fractures after a 0-10 day lag [RR (95% CI)]

**Supplementary Figure 1.** Fracture risk vs. new confirmed COVID-19 relationships by subtype in China. (A) male, (B) female, (C) children, (D) young men, (E) young women, (F) elderly men, (G) elderly women, (H) regions with >1000 cumulative confirmed cases, (I) regions with 500-1000 cumulative confirmed cases, (J) regions with <500 cumulative confirmed cases, (K) limb fracture, (L) trunk fracture, (M) single fracture, (N) multiple fractures, (O) low-energy fracture, and (P) high-energy fracture. Note: A1-P1 show fractures in three-dimensional graphs, A2-P2 demonstrate fractures in two-dimensional contour plots, and A3-P3 show fractures in two-dimensional graphs.
