# Supplementary figures and images for: Short-term effects of COVID-19 on the risk of traumatic fractures in China cities
Source: Sci Rep. 2022 Apr 20;12:6528. doi: 10.1038/s41598-022-10531-2 (PMC9020760; doi:10.1038/s41598-022-10531-2)

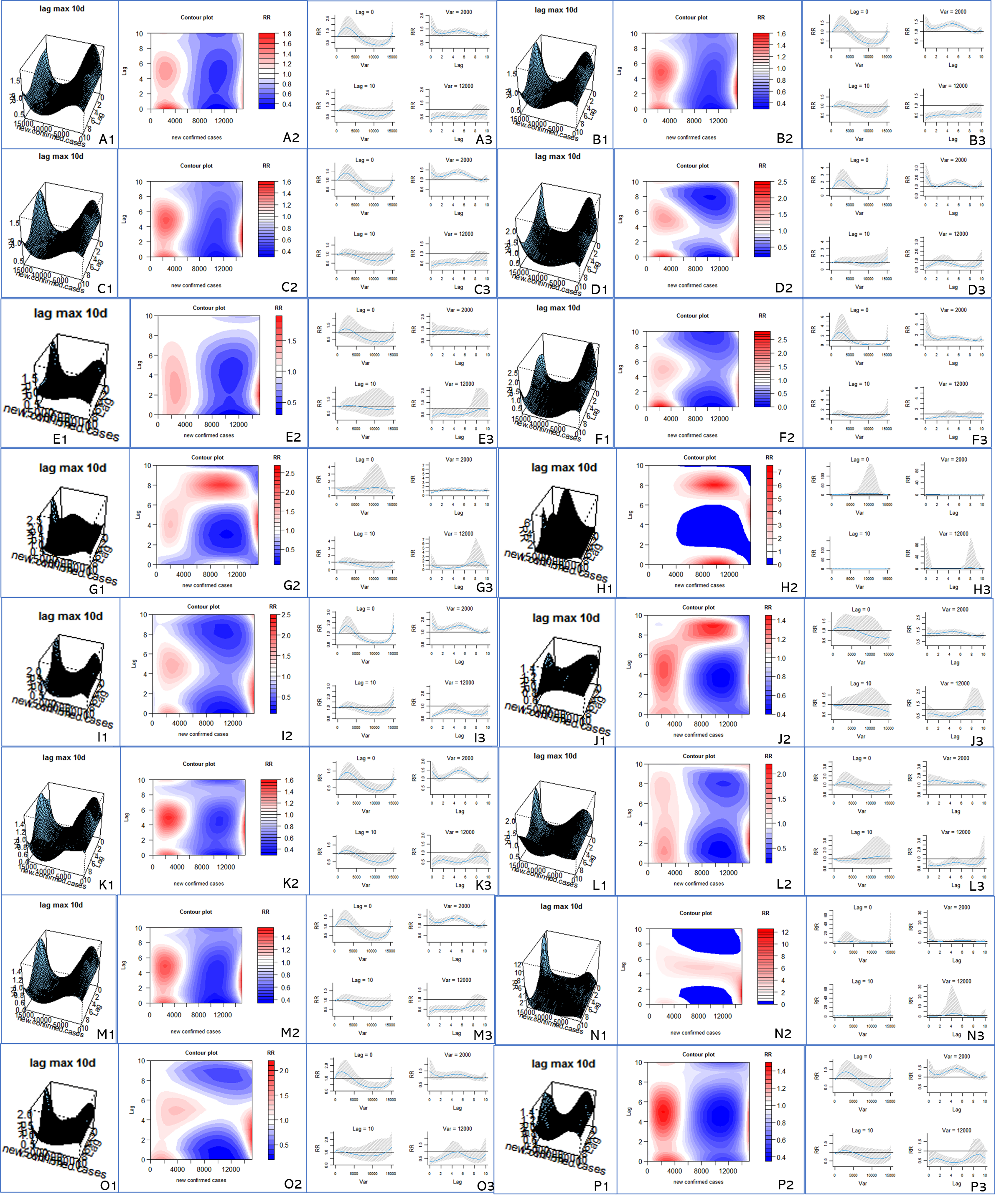

Supplement: Supplementary file 2 — Supplementary Figure 1. [file 41598_2022_10531_MOESM2_ESM.tif]
