# Supplementary material for: Short-term effects of COVID-19 on the risk of traumatic fractures in China cities
Source: Sci Rep. 2022 Apr 20;12:6528. doi: 10.1038/s41598-022-10531-2 (PMC9020760; doi:10.1038/s41598-022-10531-2)
Supplement: Supplementary file 3 — Supplementary Information. [file 41598_2022_10531_MOESM3_ESM.docx]

**Supplementary File 1.** Management principle for traumatic fracture in the epidemic of COVID-19

To ensure that COVID-19 confirmed or suspected cases, or common fractures, were diagnosed in every patient, the recent epidemiological history of the patient was confirmed. At the same time, necessary laboratory and imaging examinations were performed, such as routine blood tests, C-reactive protein, erythrocyte sedimentation rate, lung CT, and viral nucleic acid detection of throat swabs and respiratory tract secretions. All patients with common fractures were treated with first-class protective measures, strict preoperative preparation, intraoperative treatment principles, postoperative nursing and rehabilitation, and follow-up after discharge, so as to formulate a meticulous and operable plan. If COVID-19 was diagnosed or suspected, the isolation measures were immediately implemented and upgraded to level three protection. At the same time, the orthopedics department; infection department; respiratory department; intensive care unit; anesthesiology department; digestive department; and other relevant departments were immediately organized for consultation, and the precise treatment concept of “one person, one policy” was implemented. In principle, patients treatable with a brace were not operated on. For patients requiring surgery, we chose minimally invasive treatment under the premise of ensuring the quality of fracture reduction, so as to decrease the secondary impact of surgical trauma on the immunity of patients. According to the changes in the disease, the treatment plan was adjustable at any time.
