# Supplementary material for: Short-term effects of COVID-19 on the risk of traumatic fractures in China cities
Source: Sci Rep. 2022 Apr 20;12:6528. doi: 10.1038/s41598-022-10531-2 (PMC9020760; doi:10.1038/s41598-022-10531-2)
Supplement: Supplementary file 4 — Supplementary Table 1. [file 41598_2022_10531_MOESM4_ESM.docx]

**Supplementary** **Table 1**. Effects of new confirmed COVID-19 on traumatic fractures after a 0-10 day lag [RR(95%CI)]

| new confirmed COVID-19 | lag0 | | | |  | lag1 | | | |  | lag2 | | | |  | lag3 | | | |  | lag4 | | | |  | lag5 | | | |
| --- | --- | --- | --- | --- | --- | --- | --- | --- | --- | --- | --- | --- | --- | --- | --- | --- | --- | --- | --- | --- | --- | --- | --- | --- | --- | --- | --- | --- | --- |
| 0 | 1 | 1 | ~ | 1 |  | 1 | 1 | ~ | 1 |  | 1 | 1 | ~ | 1 |  | 1 | 1 | ~ | 1 |  | 1 | 1 | ~ | 1 |  | 1 | 1 | ~ | 1 |
| 100 | 1.05 | 0.95 | ~ | 1.05 |  | 0.98 | 0.92 | ~ | 0.98 |  | 0.98 | 0.93 | ~ | 0.98 |  | 1.01 | 0.96 | ~ | 1.01 |  | 1.04 | 0.99 | ~ | 1.04 |  | 1.07 | 1.01 | ~ | 1.07 |
| 200 | 1.09 | 0.97 | ~ | 1.09 |  | 1.00 | 0.94 | ~ | 1.00 |  | 1.01 | 0.94 | ~ | 1.01 |  | 1.04 | 0.98 | ~ | 1.04 |  | 1.07 | 1.02 | ~ | 1.07 |  | 1.10 | 1.04 | ~ | 1.10 |
| 300 | 1.13 | 0.98 | ~ | 1.13 |  | 1.03 | 0.95 | ~ | 1.03 |  | 1.03 | 0.95 | ~ | 1.03 |  | 1.06 | 1.00 | ~ | 1.06 |  | 1.11 | 1.04 | ~ | 1.11 |  | 1.13 | 1.06 | ~ | 1.13 |
| 400 | 1.18 | 1.00 | ~ | 1.18 |  | 1.05 | 0.96 | ~ | 1.05 |  | 1.06 | 0.96 | ~ | 1.06 |  | 1.09 | 1.01 | ~ | 1.09 |  | 1.14 | 1.06 | ~ | 1.14 |  | 1.17 | 1.08 | ~ | 1.17 |
| 500 | 1.23 | 1.01 | ~ | 1.23 |  | 1.08 | 0.97 | ~ | 1.08 |  | 1.09 | 0.97 | ~ | 1.09 |  | 1.12 | 1.03 | ~ | 1.12 |  | 1.17 | 1.08 | ~ | 1.17 |  | 1.20 | 1.10 | ~ | 1.20 |
| 600 | 1.28 | 1.02 | ~ | 1.28 |  | 1.11 | 0.97 | ~ | 1.11 |  | 1.11 | 0.98 | ~ | 1.11 |  | 1.15 | 1.04 | ~ | 1.15 |  | 1.21 | 1.10 | ~ | 1.21 |  | 1.24 | 1.12 | ~ | 1.24 |
| 700 | 1.33 | 1.03 | ~ | 1.33 |  | 1.13 | 0.98 | ~ | 1.13 |  | 1.14 | 0.99 | ~ | 1.14 |  | 1.18 | 1.06 | ~ | 1.18 |  | 1.24 | 1.11 | ~ | 1.24 |  | 1.27 | 1.13 | ~ | 1.27 |
| 800 | 1.38 | 1.03 | ~ | 1.38 |  | 1.15 | 0.98 | ~ | 1.15 |  | 1.16 | 0.99 | ~ | 1.16 |  | 1.20 | 1.07 | ~ | 1.20 |  | 1.27 | 1.13 | ~ | 1.27 |  | 1.30 | 1.15 | ~ | 1.30 |
| 900 | 1.43 | 1.04 | ~ | 1.43 |  | 1.18 | 0.99 | ~ | 1.18 |  | 1.18 | 1.00 | ~ | 1.18 |  | 1.23 | 1.08 | ~ | 1.23 |  | 1.30 | 1.15 | ~ | 1.30 |  | 1.33 | 1.16 | ~ | 1.33 |
| 1000 | 1.47 | 1.04 | ~ | 1.47 |  | 1.20 | 0.99 | ~ | 1.20 |  | 1.20 | 1.00 | ~ | 1.20 |  | 1.25 | 1.09 | ~ | 1.25 |  | 1.33 | 1.16 | ~ | 1.33 |  | 1.36 | 1.17 | ~ | 1.36 |
| 1100 | 1.52 | 1.05 | ~ | 1.52 |  | 1.22 | 0.99 | ~ | 1.22 |  | 1.22 | 1.00 | ~ | 1.22 |  | 1.27 | 1.10 | ~ | 1.27 |  | 1.35 | 1.17 | ~ | 1.35 |  | 1.39 | 1.19 | ~ | 1.39 |
| 1200 | 1.56 | 1.05 | ~ | 1.56 |  | 1.24 | 1.00 | ~ | 1.24 |  | 1.24 | 1.01 | ~ | 1.24 |  | 1.29 | 1.11 | ~ | 1.29 |  | 1.38 | 1.19 | ~ | 1.38 |  | 1.42 | 1.20 | ~ | 1.42 |
| 1300 | 1.60 | 1.06 | ~ | 1.60 |  | 1.25 | 1.00 | ~ | 1.25 |  | 1.26 | 1.01 | ~ | 1.26 |  | 1.31 | 1.12 | ~ | 1.31 |  | 1.40 | 1.20 | ~ | 1.40 |  | 1.44 | 1.21 | ~ | 1.44 |
| 1400 | 1.64 | 1.06 | ~ | 1.64 |  | 1.27 | 1.00 | ~ | 1.27 |  | 1.28 | 1.01 | ~ | 1.28 |  | 1.33 | 1.12 | ~ | 1.33 |  | 1.42 | 1.21 | ~ | 1.42 |  | 1.46 | 1.22 | ~ | 1.46 |
| 1500 | 1.67 | 1.06 | ~ | 1.67 |  | 1.28 | 1.00 | ~ | 1.28 |  | 1.29 | 1.01 | ~ | 1.29 |  | 1.35 | 1.13 | ~ | 1.35 |  | 1.44 | 1.22 | ~ | 1.44 |  | 1.49 | 1.23 | ~ | 1.49 |
| 1600 | 1.70 | 1.06 | ~ | 1.70 |  | 1.30 | 1.00 | ~ | 1.30 |  | 1.30 | 1.01 | ~ | 1.30 |  | 1.36 | 1.13 | ~ | 1.36 |  | 1.46 | 1.22 | ~ | 1.46 |  | 1.51 | 1.24 | ~ | 1.51 |
| 1700 | 1.73 | 1.06 | ~ | 1.73 |  | 1.31 | 1.00 | ~ | 1.31 |  | 1.32 | 1.01 | ~ | 1.32 |  | 1.38 | 1.14 | ~ | 1.38 |  | 1.48 | 1.23 | ~ | 1.48 |  | 1.52 | 1.24 | ~ | 1.52 |
| 1800 | 1.76 | 1.06 | ~ | 1.76 |  | 1.32 | 1.00 | ~ | 1.32 |  | 1.33 | 1.01 | ~ | 1.33 |  | 1.39 | 1.14 | ~ | 1.39 |  | 1.49 | 1.24 | ~ | 1.49 |  | 1.54 | 1.25 | ~ | 1.54 |
| 1900 | 1.79 | 1.06 | ~ | 1.79 |  | 1.33 | 0.99 | ~ | 1.33 |  | 1.34 | 1.01 | ~ | 1.34 |  | 1.40 | 1.14 | ~ | 1.40 |  | 1.51 | 1.24 | ~ | 1.51 |  | 1.56 | 1.25 | ~ | 1.56 |
| 2000 | 1.81 | 1.05 | ~ | 1.81 |  | 1.33 | 0.99 | ~ | 1.33 |  | 1.34 | 1.01 | ~ | 1.34 |  | 1.41 | 1.14 | ~ | 1.41 |  | 1.52 | 1.25 | ~ | 1.52 |  | 1.57 | 1.26 | ~ | 1.57 |
| 2100 | 1.83 | 1.05 | ~ | 1.83 |  | 1.34 | 0.99 | ~ | 1.34 |  | 1.35 | 1.00 | ~ | 1.35 |  | 1.41 | 1.14 | ~ | 1.41 |  | 1.53 | 1.25 | ~ | 1.53 |  | 1.58 | 1.26 | ~ | 1.58 |
| 2200 | 1.84 | 1.05 | ~ | 1.84 |  | 1.34 | 0.98 | ~ | 1.34 |  | 1.35 | 1.00 | ~ | 1.35 |  | 1.42 | 1.14 | ~ | 1.42 |  | 1.53 | 1.25 | ~ | 1.53 |  | 1.59 | 1.26 | ~ | 1.59 |
| 2300 | 1.86 | 1.04 | ~ | 1.86 |  | 1.34 | 0.98 | ~ | 1.34 |  | 1.36 | 1.00 | ~ | 1.36 |  | 1.42 | 1.14 | ~ | 1.42 |  | 1.54 | 1.25 | ~ | 1.54 |  | 1.60 | 1.26 | ~ | 1.60 |
| 2400 | 1.87 | 1.04 | ~ | 1.87 |  | 1.34 | 0.98 | ~ | 1.34 |  | 1.36 | 0.99 | ~ | 1.36 |  | 1.43 | 1.14 | ~ | 1.43 |  | 1.55 | 1.25 | ~ | 1.55 |  | 1.61 | 1.26 | ~ | 1.61 |
| 2500 | 1.87 | 1.03 | ~ | 1.87 |  | 1.34 | 0.97 | ~ | 1.34 |  | 1.36 | 0.99 | ~ | 1.36 |  | 1.43 | 1.14 | ~ | 1.43 |  | 1.55 | 1.25 | ~ | 1.55 |  | 1.61 | 1.26 | ~ | 1.61 |
| 2600 | 1.88 | 1.03 | ~ | 1.88 |  | 1.34 | 0.96 | ~ | 1.34 |  | 1.36 | 0.98 | ~ | 1.36 |  | 1.43 | 1.13 | ~ | 1.43 |  | 1.55 | 1.25 | ~ | 1.55 |  | 1.61 | 1.25 | ~ | 1.61 |
| 2700 | 1.88 | 1.02 | ~ | 1.88 |  | 1.34 | 0.96 | ~ | 1.34 |  | 1.35 | 0.97 | ~ | 1.35 |  | 1.43 | 1.13 | ~ | 1.43 |  | 1.55 | 1.24 | ~ | 1.55 |  | 1.62 | 1.25 | ~ | 1.62 |
| 2800 | 1.88 | 1.01 | ~ | 1.88 |  | 1.33 | 0.95 | ~ | 1.33 |  | 1.35 | 0.97 | ~ | 1.35 |  | 1.42 | 1.12 | ~ | 1.42 |  | 1.55 | 1.24 | ~ | 1.55 |  | 1.62 | 1.25 | ~ | 1.62 |
| 2900 | 1.87 | 1.00 | ~ | 1.87 |  | 1.33 | 0.94 | ~ | 1.33 |  | 1.34 | 0.96 | ~ | 1.34 |  | 1.42 | 1.12 | ~ | 1.42 |  | 1.55 | 1.23 | ~ | 1.55 |  | 1.62 | 1.24 | ~ | 1.62 |
| 3000 | 1.87 | 0.99 | ~ | 1.87 |  | 1.32 | 0.94 | ~ | 1.32 |  | 1.34 | 0.95 | ~ | 1.34 |  | 1.41 | 1.11 | ~ | 1.41 |  | 1.54 | 1.22 | ~ | 1.54 |  | 1.61 | 1.24 | ~ | 1.61 |
| 3100 | 1.86 | 0.99 | ~ | 1.86 |  | 1.31 | 0.93 | ~ | 1.31 |  | 1.33 | 0.95 | ~ | 1.33 |  | 1.41 | 1.10 | ~ | 1.41 |  | 1.54 | 1.22 | ~ | 1.54 |  | 1.61 | 1.23 | ~ | 1.61 |
| 3200 | 1.85 | 0.98 | ~ | 1.85 |  | 1.30 | 0.92 | ~ | 1.30 |  | 1.32 | 0.94 | ~ | 1.32 |  | 1.40 | 1.09 | ~ | 1.40 |  | 1.53 | 1.21 | ~ | 1.53 |  | 1.60 | 1.22 | ~ | 1.60 |
| 3300 | 1.84 | 0.97 | ~ | 1.84 |  | 1.30 | 0.91 | ~ | 1.30 |  | 1.31 | 0.93 | ~ | 1.31 |  | 1.39 | 1.08 | ~ | 1.39 |  | 1.53 | 1.20 | ~ | 1.53 |  | 1.60 | 1.21 | ~ | 1.60 |
| 3400 | 1.82 | 0.95 | ~ | 1.82 |  | 1.29 | 0.90 | ~ | 1.29 |  | 1.30 | 0.92 | ~ | 1.30 |  | 1.38 | 1.07 | ~ | 1.38 |  | 1.52 | 1.19 | ~ | 1.52 |  | 1.59 | 1.20 | ~ | 1.59 |
| 3500 | 1.81 | 0.94 | ~ | 1.81 |  | 1.28 | 0.89 | ~ | 1.28 |  | 1.29 | 0.91 | ~ | 1.29 |  | 1.37 | 1.06 | ~ | 1.37 |  | 1.51 | 1.18 | ~ | 1.51 |  | 1.58 | 1.19 | ~ | 1.58 |
| 3600 | 1.79 | 0.93 | ~ | 1.79 |  | 1.26 | 0.88 | ~ | 1.26 |  | 1.28 | 0.90 | ~ | 1.28 |  | 1.36 | 1.05 | ~ | 1.36 |  | 1.50 | 1.16 | ~ | 1.50 |  | 1.57 | 1.18 | ~ | 1.57 |
| 3700 | 1.77 | 0.92 | ~ | 1.77 |  | 1.25 | 0.87 | ~ | 1.25 |  | 1.27 | 0.89 | ~ | 1.27 |  | 1.35 | 1.04 | ~ | 1.35 |  | 1.49 | 1.15 | ~ | 1.49 |  | 1.56 | 1.17 | ~ | 1.56 |
| 3800 | 1.75 | 0.91 | ~ | 1.75 |  | 1.24 | 0.86 | ~ | 1.24 |  | 1.26 | 0.88 | ~ | 1.26 |  | 1.34 | 1.03 | ~ | 1.34 |  | 1.48 | 1.14 | ~ | 1.48 |  | 1.55 | 1.16 | ~ | 1.55 |
| 3900 | 1.73 | 0.89 | ~ | 1.73 |  | 1.23 | 0.85 | ~ | 1.23 |  | 1.25 | 0.87 | ~ | 1.25 |  | 1.33 | 1.02 | ~ | 1.33 |  | 1.47 | 1.12 | ~ | 1.47 |  | 1.54 | 1.14 | ~ | 1.54 |
| 4000 | 1.70 | 0.88 | ~ | 1.70 |  | 1.21 | 0.84 | ~ | 1.21 |  | 1.23 | 0.86 | ~ | 1.23 |  | 1.31 | 1.01 | ~ | 1.31 |  | 1.45 | 1.11 | ~ | 1.45 |  | 1.53 | 1.13 | ~ | 1.53 |
| 4100 | 1.68 | 0.87 | ~ | 1.68 |  | 1.20 | 0.83 | ~ | 1.20 |  | 1.22 | 0.85 | ~ | 1.22 |  | 1.30 | 0.99 | ~ | 1.30 |  | 1.44 | 1.09 | ~ | 1.44 |  | 1.51 | 1.11 | ~ | 1.51 |
| 4200 | 1.65 | 0.85 | ~ | 1.65 |  | 1.19 | 0.81 | ~ | 1.19 |  | 1.20 | 0.84 | ~ | 1.20 |  | 1.28 | 0.98 | ~ | 1.28 |  | 1.43 | 1.07 | ~ | 1.43 |  | 1.50 | 1.10 | ~ | 1.50 |
| 4300 | 1.63 | 0.84 | ~ | 1.63 |  | 1.17 | 0.80 | ~ | 1.17 |  | 1.19 | 0.83 | ~ | 1.19 |  | 1.27 | 0.96 | ~ | 1.27 |  | 1.42 | 1.06 | ~ | 1.42 |  | 1.49 | 1.08 | ~ | 1.49 |
| 4400 | 1.60 | 0.82 | ~ | 1.60 |  | 1.16 | 0.79 | ~ | 1.16 |  | 1.17 | 0.82 | ~ | 1.17 |  | 1.25 | 0.95 | ~ | 1.25 |  | 1.40 | 1.04 | ~ | 1.40 |  | 1.47 | 1.07 | ~ | 1.47 |
| 4500 | 1.58 | 0.81 | ~ | 1.58 |  | 1.15 | 0.77 | ~ | 1.15 |  | 1.16 | 0.81 | ~ | 1.16 |  | 1.24 | 0.94 | ~ | 1.24 |  | 1.39 | 1.02 | ~ | 1.39 |  | 1.46 | 1.05 | ~ | 1.46 |
| 4600 | 1.55 | 0.79 | ~ | 1.55 |  | 1.13 | 0.76 | ~ | 1.13 |  | 1.14 | 0.80 | ~ | 1.14 |  | 1.22 | 0.92 | ~ | 1.22 |  | 1.37 | 1.00 | ~ | 1.37 |  | 1.44 | 1.03 | ~ | 1.44 |
| 4700 | 1.52 | 0.78 | ~ | 1.52 |  | 1.12 | 0.75 | ~ | 1.12 |  | 1.13 | 0.78 | ~ | 1.13 |  | 1.21 | 0.91 | ~ | 1.21 |  | 1.36 | 0.98 | ~ | 1.36 |  | 1.42 | 1.02 | ~ | 1.42 |
| 4800 | 1.50 | 0.76 | ~ | 1.50 |  | 1.10 | 0.73 | ~ | 1.10 |  | 1.11 | 0.77 | ~ | 1.11 |  | 1.19 | 0.89 | ~ | 1.19 |  | 1.34 | 0.97 | ~ | 1.34 |  | 1.41 | 1.00 | ~ | 1.41 |
| 4900 | 1.47 | 0.75 | ~ | 1.47 |  | 1.09 | 0.72 | ~ | 1.09 |  | 1.10 | 0.76 | ~ | 1.10 |  | 1.18 | 0.87 | ~ | 1.18 |  | 1.33 | 0.95 | ~ | 1.33 |  | 1.39 | 0.98 | ~ | 1.39 |
| 5000 | 1.44 | 0.73 | ~ | 1.44 |  | 1.08 | 0.71 | ~ | 1.08 |  | 1.08 | 0.75 | ~ | 1.08 |  | 1.16 | 0.86 | ~ | 1.16 |  | 1.31 | 0.93 | ~ | 1.31 |  | 1.38 | 0.96 | ~ | 1.38 |
| 5100 | 1.42 | 0.71 | ~ | 1.42 |  | 1.06 | 0.69 | ~ | 1.06 |  | 1.07 | 0.74 | ~ | 1.07 |  | 1.15 | 0.84 | ~ | 1.15 |  | 1.30 | 0.91 | ~ | 1.30 |  | 1.36 | 0.95 | ~ | 1.36 |
| 5200 | 1.39 | 0.70 | ~ | 1.39 |  | 1.05 | 0.68 | ~ | 1.05 |  | 1.05 | 0.72 | ~ | 1.05 |  | 1.13 | 0.83 | ~ | 1.13 |  | 1.28 | 0.89 | ~ | 1.28 |  | 1.34 | 0.93 | ~ | 1.34 |
| 5300 | 1.36 | 0.68 | ~ | 1.36 |  | 1.04 | 0.67 | ~ | 1.04 |  | 1.04 | 0.71 | ~ | 1.04 |  | 1.12 | 0.81 | ~ | 1.12 |  | 1.27 | 0.87 | ~ | 1.27 |  | 1.33 | 0.91 | ~ | 1.33 |
| 5400 | 1.34 | 0.66 | ~ | 1.34 |  | 1.02 | 0.65 | ~ | 1.02 |  | 1.02 | 0.70 | ~ | 1.02 |  | 1.10 | 0.80 | ~ | 1.10 |  | 1.25 | 0.85 | ~ | 1.25 |  | 1.31 | 0.89 | ~ | 1.31 |
| 5500 | 1.31 | 0.65 | ~ | 1.31 |  | 1.01 | 0.64 | ~ | 1.01 |  | 1.01 | 0.69 | ~ | 1.01 |  | 1.09 | 0.78 | ~ | 1.09 |  | 1.24 | 0.83 | ~ | 1.24 |  | 1.29 | 0.87 | ~ | 1.29 |
| 5600 | 1.29 | 0.63 | ~ | 1.29 |  | 1.00 | 0.62 | ~ | 1.00 |  | 0.99 | 0.67 | ~ | 0.99 |  | 1.07 | 0.76 | ~ | 1.07 |  | 1.22 | 0.81 | ~ | 1.22 |  | 1.28 | 0.85 | ~ | 1.28 |
| 5700 | 1.26 | 0.62 | ~ | 1.26 |  | 0.99 | 0.61 | ~ | 0.99 |  | 0.98 | 0.66 | ~ | 0.98 |  | 1.06 | 0.75 | ~ | 1.06 |  | 1.21 | 0.79 | ~ | 1.21 |  | 1.26 | 0.84 | ~ | 1.26 |
| 5800 | 1.24 | 0.60 | ~ | 1.24 |  | 0.97 | 0.60 | ~ | 0.97 |  | 0.96 | 0.65 | ~ | 0.96 |  | 1.04 | 0.73 | ~ | 1.04 |  | 1.19 | 0.77 | ~ | 1.19 |  | 1.24 | 0.82 | ~ | 1.24 |
| 5900 | 1.21 | 0.58 | ~ | 1.21 |  | 0.96 | 0.58 | ~ | 0.96 |  | 0.95 | 0.64 | ~ | 0.95 |  | 1.03 | 0.72 | ~ | 1.03 |  | 1.18 | 0.76 | ~ | 1.18 |  | 1.23 | 0.80 | ~ | 1.23 |
| 6000 | 1.19 | 0.57 | ~ | 1.19 |  | 0.95 | 0.57 | ~ | 0.95 |  | 0.93 | 0.63 | ~ | 0.93 |  | 1.01 | 0.70 | ~ | 1.01 |  | 1.16 | 0.74 | ~ | 1.16 |  | 1.21 | 0.78 | ~ | 1.21 |
| 6100 | 1.17 | 0.55 | ~ | 1.17 |  | 0.94 | 0.56 | ~ | 0.94 |  | 0.92 | 0.61 | ~ | 0.92 |  | 1.00 | 0.69 | ~ | 1.00 |  | 1.15 | 0.72 | ~ | 1.15 |  | 1.19 | 0.77 | ~ | 1.19 |
| 6200 | 1.15 | 0.54 | ~ | 1.15 |  | 0.93 | 0.55 | ~ | 0.93 |  | 0.91 | 0.60 | ~ | 0.91 |  | 0.99 | 0.67 | ~ | 0.99 |  | 1.13 | 0.70 | ~ | 1.13 |  | 1.18 | 0.75 | ~ | 1.18 |
| 6300 | 1.12 | 0.52 | ~ | 1.12 |  | 0.91 | 0.53 | ~ | 0.91 |  | 0.89 | 0.59 | ~ | 0.89 |  | 0.97 | 0.66 | ~ | 0.97 |  | 1.12 | 0.69 | ~ | 1.12 |  | 1.16 | 0.73 | ~ | 1.16 |
| 6400 | 1.10 | 0.51 | ~ | 1.10 |  | 0.90 | 0.52 | ~ | 0.90 |  | 0.88 | 0.58 | ~ | 0.88 |  | 0.96 | 0.64 | ~ | 0.96 |  | 1.10 | 0.67 | ~ | 1.10 |  | 1.15 | 0.71 | ~ | 1.15 |
| 6500 | 1.08 | 0.49 | ~ | 1.08 |  | 0.89 | 0.51 | ~ | 0.89 |  | 0.87 | 0.57 | ~ | 0.87 |  | 0.94 | 0.63 | ~ | 0.94 |  | 1.09 | 0.65 | ~ | 1.09 |  | 1.13 | 0.70 | ~ | 1.13 |
| 6600 | 1.06 | 0.48 | ~ | 1.06 |  | 0.88 | 0.50 | ~ | 0.88 |  | 0.86 | 0.56 | ~ | 0.86 |  | 0.93 | 0.61 | ~ | 0.93 |  | 1.07 | 0.64 | ~ | 1.07 |  | 1.11 | 0.68 | ~ | 1.11 |
| 6700 | 1.04 | 0.46 | ~ | 1.04 |  | 0.87 | 0.49 | ~ | 0.87 |  | 0.85 | 0.54 | ~ | 0.85 |  | 0.92 | 0.60 | ~ | 0.92 |  | 1.06 | 0.62 | ~ | 1.06 |  | 1.10 | 0.67 | ~ | 1.10 |
| 6800 | 1.03 | 0.45 | ~ | 1.03 |  | 0.86 | 0.47 | ~ | 0.86 |  | 0.84 | 0.53 | ~ | 0.84 |  | 0.91 | 0.59 | ~ | 0.91 |  | 1.05 | 0.60 | ~ | 1.05 |  | 1.08 | 0.65 | ~ | 1.08 |
| 6900 | 1.01 | 0.44 | ~ | 1.01 |  | 0.85 | 0.46 | ~ | 0.85 |  | 0.82 | 0.52 | ~ | 0.82 |  | 0.89 | 0.57 | ~ | 0.89 |  | 1.03 | 0.59 | ~ | 1.03 |  | 1.07 | 0.64 | ~ | 1.07 |
| 7000 | 0.99 | 0.42 | ~ | 0.99 |  | 0.84 | 0.45 | ~ | 0.84 |  | 0.81 | 0.51 | ~ | 0.81 |  | 0.88 | 0.56 | ~ | 0.88 |  | 1.02 | 0.57 | ~ | 1.02 |  | 1.05 | 0.62 | ~ | 1.05 |
| 7100 | 0.97 | 0.41 | ~ | 0.97 |  | 0.83 | 0.44 | ~ | 0.83 |  | 0.80 | 0.50 | ~ | 0.80 |  | 0.87 | 0.55 | ~ | 0.87 |  | 1.01 | 0.56 | ~ | 1.01 |  | 1.04 | 0.61 | ~ | 1.04 |
| 7200 | 0.96 | 0.40 | ~ | 0.96 |  | 0.82 | 0.43 | ~ | 0.82 |  | 0.79 | 0.49 | ~ | 0.79 |  | 0.86 | 0.54 | ~ | 0.86 |  | 0.99 | 0.55 | ~ | 0.99 |  | 1.03 | 0.59 | ~ | 1.03 |
| 7300 | 0.94 | 0.39 | ~ | 0.94 |  | 0.81 | 0.42 | ~ | 0.81 |  | 0.78 | 0.48 | ~ | 0.78 |  | 0.85 | 0.53 | ~ | 0.85 |  | 0.98 | 0.53 | ~ | 0.98 |  | 1.01 | 0.58 | ~ | 1.01 |
| 7400 | 0.92 | 0.37 | ~ | 0.92 |  | 0.80 | 0.41 | ~ | 0.80 |  | 0.77 | 0.47 | ~ | 0.77 |  | 0.84 | 0.51 | ~ | 0.84 |  | 0.97 | 0.52 | ~ | 0.97 |  | 1.00 | 0.57 | ~ | 1.00 |
| 7500 | 0.91 | 0.36 | ~ | 0.91 |  | 0.79 | 0.40 | ~ | 0.79 |  | 0.76 | 0.47 | ~ | 0.76 |  | 0.83 | 0.50 | ~ | 0.83 |  | 0.96 | 0.51 | ~ | 0.96 |  | 0.98 | 0.55 | ~ | 0.98 |
| 7600 | 0.89 | 0.35 | ~ | 0.89 |  | 0.79 | 0.40 | ~ | 0.79 |  | 0.76 | 0.46 | ~ | 0.76 |  | 0.82 | 0.49 | ~ | 0.82 |  | 0.95 | 0.50 | ~ | 0.95 |  | 0.97 | 0.54 | ~ | 0.97 |
| 7700 | 0.88 | 0.34 | ~ | 0.88 |  | 0.78 | 0.39 | ~ | 0.78 |  | 0.75 | 0.45 | ~ | 0.75 |  | 0.81 | 0.48 | ~ | 0.81 |  | 0.93 | 0.48 | ~ | 0.93 |  | 0.96 | 0.53 | ~ | 0.96 |
| 7800 | 0.87 | 0.33 | ~ | 0.87 |  | 0.77 | 0.38 | ~ | 0.77 |  | 0.74 | 0.44 | ~ | 0.74 |  | 0.80 | 0.47 | ~ | 0.80 |  | 0.92 | 0.47 | ~ | 0.92 |  | 0.95 | 0.52 | ~ | 0.95 |
| 7900 | 0.85 | 0.32 | ~ | 0.85 |  | 0.76 | 0.37 | ~ | 0.76 |  | 0.73 | 0.43 | ~ | 0.73 |  | 0.79 | 0.46 | ~ | 0.79 |  | 0.91 | 0.46 | ~ | 0.91 |  | 0.93 | 0.51 | ~ | 0.93 |
| 8000 | 0.84 | 0.31 | ~ | 0.84 |  | 0.75 | 0.36 | ~ | 0.75 |  | 0.72 | 0.42 | ~ | 0.72 |  | 0.78 | 0.45 | ~ | 0.78 |  | 0.90 | 0.45 | ~ | 0.90 |  | 0.92 | 0.50 | ~ | 0.92 |
| 8100 | 0.83 | 0.30 | ~ | 0.83 |  | 0.75 | 0.36 | ~ | 0.75 |  | 0.72 | 0.42 | ~ | 0.72 |  | 0.77 | 0.44 | ~ | 0.77 |  | 0.89 | 0.44 | ~ | 0.89 |  | 0.91 | 0.48 | ~ | 0.91 |
| 8200 | 0.81 | 0.29 | ~ | 0.81 |  | 0.74 | 0.35 | ~ | 0.74 |  | 0.71 | 0.41 | ~ | 0.71 |  | 0.76 | 0.44 | ~ | 0.76 |  | 0.88 | 0.43 | ~ | 0.88 |  | 0.90 | 0.47 | ~ | 0.90 |
| 8300 | 0.80 | 0.29 | ~ | 0.80 |  | 0.73 | 0.34 | ~ | 0.73 |  | 0.70 | 0.40 | ~ | 0.70 |  | 0.76 | 0.43 | ~ | 0.76 |  | 0.87 | 0.42 | ~ | 0.87 |  | 0.89 | 0.47 | ~ | 0.89 |
| 8400 | 0.79 | 0.28 | ~ | 0.79 |  | 0.73 | 0.34 | ~ | 0.73 |  | 0.69 | 0.40 | ~ | 0.69 |  | 0.75 | 0.42 | ~ | 0.75 |  | 0.86 | 0.41 | ~ | 0.86 |  | 0.88 | 0.46 | ~ | 0.88 |
| 8500 | 0.78 | 0.27 | ~ | 0.78 |  | 0.72 | 0.33 | ~ | 0.72 |  | 0.69 | 0.39 | ~ | 0.69 |  | 0.74 | 0.41 | ~ | 0.74 |  | 0.85 | 0.41 | ~ | 0.85 |  | 0.87 | 0.45 | ~ | 0.87 |
| 8600 | 0.77 | 0.26 | ~ | 0.77 |  | 0.71 | 0.33 | ~ | 0.71 |  | 0.68 | 0.39 | ~ | 0.68 |  | 0.73 | 0.41 | ~ | 0.73 |  | 0.84 | 0.40 | ~ | 0.84 |  | 0.86 | 0.44 | ~ | 0.86 |
| 8700 | 0.76 | 0.26 | ~ | 0.76 |  | 0.71 | 0.32 | ~ | 0.71 |  | 0.68 | 0.38 | ~ | 0.68 |  | 0.73 | 0.40 | ~ | 0.73 |  | 0.83 | 0.39 | ~ | 0.83 |  | 0.85 | 0.43 | ~ | 0.85 |
| 8800 | 0.75 | 0.25 | ~ | 0.75 |  | 0.70 | 0.31 | ~ | 0.70 |  | 0.67 | 0.37 | ~ | 0.67 |  | 0.72 | 0.39 | ~ | 0.72 |  | 0.83 | 0.38 | ~ | 0.83 |  | 0.84 | 0.42 | ~ | 0.84 |
| 8900 | 0.74 | 0.24 | ~ | 0.74 |  | 0.70 | 0.31 | ~ | 0.70 |  | 0.67 | 0.37 | ~ | 0.67 |  | 0.72 | 0.39 | ~ | 0.72 |  | 0.82 | 0.38 | ~ | 0.82 |  | 0.83 | 0.42 | ~ | 0.83 |
| 9000 | 0.73 | 0.24 | ~ | 0.73 |  | 0.69 | 0.31 | ~ | 0.69 |  | 0.66 | 0.37 | ~ | 0.66 |  | 0.71 | 0.38 | ~ | 0.71 |  | 0.81 | 0.37 | ~ | 0.81 |  | 0.82 | 0.41 | ~ | 0.82 |
| 9100 | 0.72 | 0.23 | ~ | 0.72 |  | 0.69 | 0.30 | ~ | 0.69 |  | 0.66 | 0.36 | ~ | 0.66 |  | 0.70 | 0.37 | ~ | 0.70 |  | 0.80 | 0.36 | ~ | 0.80 |  | 0.81 | 0.40 | ~ | 0.81 |
| 9200 | 0.71 | 0.23 | ~ | 0.71 |  | 0.68 | 0.30 | ~ | 0.68 |  | 0.65 | 0.36 | ~ | 0.65 |  | 0.70 | 0.37 | ~ | 0.70 |  | 0.80 | 0.36 | ~ | 0.80 |  | 0.80 | 0.40 | ~ | 0.80 |
| 9300 | 0.71 | 0.22 | ~ | 0.71 |  | 0.68 | 0.29 | ~ | 0.68 |  | 0.65 | 0.35 | ~ | 0.65 |  | 0.70 | 0.36 | ~ | 0.70 |  | 0.79 | 0.35 | ~ | 0.79 |  | 0.79 | 0.39 | ~ | 0.79 |
| 9400 | 0.70 | 0.22 | ~ | 0.70 |  | 0.67 | 0.29 | ~ | 0.67 |  | 0.65 | 0.35 | ~ | 0.65 |  | 0.69 | 0.36 | ~ | 0.69 |  | 0.78 | 0.35 | ~ | 0.78 |  | 0.79 | 0.38 | ~ | 0.79 |
| 9500 | 0.69 | 0.21 | ~ | 0.69 |  | 0.67 | 0.29 | ~ | 0.67 |  | 0.64 | 0.35 | ~ | 0.64 |  | 0.69 | 0.36 | ~ | 0.69 |  | 0.78 | 0.34 | ~ | 0.78 |  | 0.78 | 0.38 | ~ | 0.78 |
| 9600 | 0.68 | 0.21 | ~ | 0.68 |  | 0.66 | 0.29 | ~ | 0.66 |  | 0.64 | 0.34 | ~ | 0.64 |  | 0.68 | 0.35 | ~ | 0.68 |  | 0.77 | 0.34 | ~ | 0.77 |  | 0.77 | 0.37 | ~ | 0.77 |
| 9700 | 0.68 | 0.21 | ~ | 0.68 |  | 0.66 | 0.28 | ~ | 0.66 |  | 0.64 | 0.34 | ~ | 0.64 |  | 0.68 | 0.35 | ~ | 0.68 |  | 0.77 | 0.33 | ~ | 0.77 |  | 0.77 | 0.37 | ~ | 0.77 |
| 9800 | 0.67 | 0.20 | ~ | 0.67 |  | 0.66 | 0.28 | ~ | 0.66 |  | 0.64 | 0.34 | ~ | 0.64 |  | 0.68 | 0.35 | ~ | 0.68 |  | 0.76 | 0.33 | ~ | 0.76 |  | 0.76 | 0.37 | ~ | 0.76 |
| 9900 | 0.66 | 0.20 | ~ | 0.66 |  | 0.65 | 0.28 | ~ | 0.65 |  | 0.63 | 0.34 | ~ | 0.63 |  | 0.68 | 0.34 | ~ | 0.68 |  | 0.76 | 0.33 | ~ | 0.76 |  | 0.75 | 0.36 | ~ | 0.75 |
| 10000 | 0.66 | 0.20 | ~ | 0.66 |  | 0.65 | 0.28 | ~ | 0.65 |  | 0.63 | 0.33 | ~ | 0.63 |  | 0.67 | 0.34 | ~ | 0.67 |  | 0.75 | 0.32 | ~ | 0.75 |  | 0.75 | 0.36 | ~ | 0.75 |
| 10100 | 0.65 | 0.19 | ~ | 0.65 |  | 0.65 | 0.28 | ~ | 0.65 |  | 0.63 | 0.33 | ~ | 0.63 |  | 0.67 | 0.34 | ~ | 0.67 |  | 0.75 | 0.32 | ~ | 0.75 |  | 0.74 | 0.36 | ~ | 0.74 |
| 10200 | 0.65 | 0.19 | ~ | 0.65 |  | 0.65 | 0.28 | ~ | 0.65 |  | 0.63 | 0.33 | ~ | 0.63 |  | 0.67 | 0.34 | ~ | 0.67 |  | 0.75 | 0.32 | ~ | 0.75 |  | 0.74 | 0.35 | ~ | 0.74 |
| 10300 | 0.64 | 0.19 | ~ | 0.64 |  | 0.64 | 0.28 | ~ | 0.64 |  | 0.63 | 0.33 | ~ | 0.63 |  | 0.67 | 0.33 | ~ | 0.67 |  | 0.74 | 0.32 | ~ | 0.74 |  | 0.73 | 0.35 | ~ | 0.73 |
| 10400 | 0.64 | 0.19 | ~ | 0.64 |  | 0.64 | 0.27 | ~ | 0.64 |  | 0.63 | 0.33 | ~ | 0.63 |  | 0.67 | 0.33 | ~ | 0.67 |  | 0.74 | 0.32 | ~ | 0.74 |  | 0.73 | 0.35 | ~ | 0.73 |
| 10500 | 0.63 | 0.19 | ~ | 0.63 |  | 0.64 | 0.27 | ~ | 0.64 |  | 0.63 | 0.33 | ~ | 0.63 |  | 0.67 | 0.33 | ~ | 0.67 |  | 0.74 | 0.31 | ~ | 0.74 |  | 0.73 | 0.35 | ~ | 0.73 |
| 10600 | 0.63 | 0.19 | ~ | 0.63 |  | 0.64 | 0.28 | ~ | 0.64 |  | 0.63 | 0.33 | ~ | 0.63 |  | 0.67 | 0.33 | ~ | 0.67 |  | 0.74 | 0.31 | ~ | 0.74 |  | 0.72 | 0.35 | ~ | 0.72 |
| 10700 | 0.63 | 0.19 | ~ | 0.63 |  | 0.64 | 0.28 | ~ | 0.64 |  | 0.63 | 0.33 | ~ | 0.63 |  | 0.67 | 0.33 | ~ | 0.67 |  | 0.74 | 0.31 | ~ | 0.74 |  | 0.72 | 0.34 | ~ | 0.72 |
| 10800 | 0.62 | 0.18 | ~ | 0.62 |  | 0.64 | 0.28 | ~ | 0.64 |  | 0.63 | 0.33 | ~ | 0.63 |  | 0.67 | 0.33 | ~ | 0.67 |  | 0.74 | 0.31 | ~ | 0.74 |  | 0.72 | 0.34 | ~ | 0.72 |
| 10900 | 0.62 | 0.18 | ~ | 0.62 |  | 0.64 | 0.28 | ~ | 0.64 |  | 0.63 | 0.34 | ~ | 0.63 |  | 0.67 | 0.33 | ~ | 0.67 |  | 0.74 | 0.31 | ~ | 0.74 |  | 0.71 | 0.34 | ~ | 0.71 |
| 11000 | 0.62 | 0.18 | ~ | 0.62 |  | 0.64 | 0.28 | ~ | 0.64 |  | 0.64 | 0.34 | ~ | 0.64 |  | 0.68 | 0.33 | ~ | 0.68 |  | 0.74 | 0.31 | ~ | 0.74 |  | 0.71 | 0.34 | ~ | 0.71 |
| 11100 | 0.61 | 0.18 | ~ | 0.61 |  | 0.64 | 0.28 | ~ | 0.64 |  | 0.64 | 0.34 | ~ | 0.64 |  | 0.68 | 0.34 | ~ | 0.68 |  | 0.74 | 0.32 | ~ | 0.74 |  | 0.71 | 0.35 | ~ | 0.71 |
| 11200 | 0.61 | 0.19 | ~ | 0.61 |  | 0.64 | 0.29 | ~ | 0.64 |  | 0.64 | 0.34 | ~ | 0.64 |  | 0.68 | 0.34 | ~ | 0.68 |  | 0.74 | 0.32 | ~ | 0.74 |  | 0.71 | 0.35 | ~ | 0.71 |
| 11300 | 0.61 | 0.19 | ~ | 0.61 |  | 0.64 | 0.29 | ~ | 0.64 |  | 0.65 | 0.35 | ~ | 0.65 |  | 0.69 | 0.34 | ~ | 0.69 |  | 0.74 | 0.32 | ~ | 0.74 |  | 0.71 | 0.35 | ~ | 0.71 |
| 11400 | 0.61 | 0.19 | ~ | 0.61 |  | 0.64 | 0.29 | ~ | 0.64 |  | 0.65 | 0.35 | ~ | 0.65 |  | 0.69 | 0.34 | ~ | 0.69 |  | 0.74 | 0.32 | ~ | 0.74 |  | 0.71 | 0.35 | ~ | 0.71 |
| 11500 | 0.61 | 0.19 | ~ | 0.61 |  | 0.64 | 0.30 | ~ | 0.64 |  | 0.65 | 0.35 | ~ | 0.65 |  | 0.70 | 0.35 | ~ | 0.70 |  | 0.74 | 0.32 | ~ | 0.74 |  | 0.71 | 0.35 | ~ | 0.71 |
| 11600 | 0.61 | 0.19 | ~ | 0.61 |  | 0.64 | 0.30 | ~ | 0.64 |  | 0.66 | 0.36 | ~ | 0.66 |  | 0.70 | 0.35 | ~ | 0.70 |  | 0.75 | 0.33 | ~ | 0.75 |  | 0.71 | 0.36 | ~ | 0.71 |
| 11700 | 0.61 | 0.19 | ~ | 0.61 |  | 0.64 | 0.31 | ~ | 0.64 |  | 0.67 | 0.36 | ~ | 0.67 |  | 0.71 | 0.35 | ~ | 0.71 |  | 0.75 | 0.33 | ~ | 0.75 |  | 0.71 | 0.36 | ~ | 0.71 |
| 11800 | 0.61 | 0.20 | ~ | 0.61 |  | 0.65 | 0.31 | ~ | 0.65 |  | 0.67 | 0.37 | ~ | 0.67 |  | 0.71 | 0.36 | ~ | 0.71 |  | 0.75 | 0.34 | ~ | 0.75 |  | 0.71 | 0.36 | ~ | 0.71 |
| 11900 | 0.61 | 0.20 | ~ | 0.61 |  | 0.65 | 0.32 | ~ | 0.65 |  | 0.68 | 0.37 | ~ | 0.68 |  | 0.72 | 0.36 | ~ | 0.72 |  | 0.76 | 0.34 | ~ | 0.76 |  | 0.72 | 0.37 | ~ | 0.72 |
| 12000 | 0.61 | 0.20 | ~ | 0.61 |  | 0.65 | 0.33 | ~ | 0.65 |  | 0.69 | 0.38 | ~ | 0.69 |  | 0.73 | 0.37 | ~ | 0.73 |  | 0.76 | 0.35 | ~ | 0.76 |  | 0.72 | 0.37 | ~ | 0.72 |
| 12100 | 0.61 | 0.21 | ~ | 0.61 |  | 0.66 | 0.33 | ~ | 0.66 |  | 0.70 | 0.39 | ~ | 0.70 |  | 0.74 | 0.38 | ~ | 0.74 |  | 0.77 | 0.35 | ~ | 0.77 |  | 0.72 | 0.38 | ~ | 0.72 |
| 12200 | 0.61 | 0.21 | ~ | 0.61 |  | 0.66 | 0.34 | ~ | 0.66 |  | 0.70 | 0.40 | ~ | 0.70 |  | 0.75 | 0.38 | ~ | 0.75 |  | 0.78 | 0.36 | ~ | 0.78 |  | 0.72 | 0.38 | ~ | 0.72 |
| 12300 | 0.61 | 0.22 | ~ | 0.61 |  | 0.67 | 0.35 | ~ | 0.67 |  | 0.71 | 0.41 | ~ | 0.71 |  | 0.76 | 0.39 | ~ | 0.76 |  | 0.78 | 0.37 | ~ | 0.78 |  | 0.73 | 0.39 | ~ | 0.73 |
| 12400 | 0.62 | 0.22 | ~ | 0.62 |  | 0.67 | 0.36 | ~ | 0.67 |  | 0.73 | 0.41 | ~ | 0.73 |  | 0.77 | 0.40 | ~ | 0.77 |  | 0.79 | 0.38 | ~ | 0.79 |  | 0.73 | 0.40 | ~ | 0.73 |
| 12500 | 0.62 | 0.23 | ~ | 0.62 |  | 0.68 | 0.37 | ~ | 0.68 |  | 0.74 | 0.43 | ~ | 0.74 |  | 0.79 | 0.41 | ~ | 0.79 |  | 0.80 | 0.39 | ~ | 0.80 |  | 0.74 | 0.41 | ~ | 0.74 |
| 12600 | 0.62 | 0.23 | ~ | 0.62 |  | 0.69 | 0.38 | ~ | 0.69 |  | 0.75 | 0.44 | ~ | 0.75 |  | 0.80 | 0.42 | ~ | 0.80 |  | 0.81 | 0.40 | ~ | 0.81 |  | 0.75 | 0.42 | ~ | 0.75 |
| 12700 | 0.63 | 0.24 | ~ | 0.63 |  | 0.69 | 0.39 | ~ | 0.69 |  | 0.77 | 0.45 | ~ | 0.77 |  | 0.82 | 0.43 | ~ | 0.82 |  | 0.82 | 0.41 | ~ | 0.82 |  | 0.75 | 0.43 | ~ | 0.75 |
| 12800 | 0.63 | 0.25 | ~ | 0.63 |  | 0.70 | 0.41 | ~ | 0.70 |  | 0.78 | 0.46 | ~ | 0.78 |  | 0.83 | 0.44 | ~ | 0.83 |  | 0.83 | 0.42 | ~ | 0.83 |  | 0.76 | 0.44 | ~ | 0.76 |
| 12900 | 0.64 | 0.26 | ~ | 0.64 |  | 0.71 | 0.42 | ~ | 0.71 |  | 0.80 | 0.47 | ~ | 0.80 |  | 0.85 | 0.46 | ~ | 0.85 |  | 0.85 | 0.43 | ~ | 0.85 |  | 0.77 | 0.45 | ~ | 0.77 |
| 13000 | 0.64 | 0.27 | ~ | 0.64 |  | 0.72 | 0.44 | ~ | 0.72 |  | 0.82 | 0.49 | ~ | 0.82 |  | 0.87 | 0.47 | ~ | 0.87 |  | 0.86 | 0.45 | ~ | 0.86 |  | 0.78 | 0.46 | ~ | 0.78 |
| 13100 | 0.65 | 0.28 | ~ | 0.65 |  | 0.73 | 0.46 | ~ | 0.73 |  | 0.84 | 0.50 | ~ | 0.84 |  | 0.89 | 0.49 | ~ | 0.89 |  | 0.88 | 0.46 | ~ | 0.88 |  | 0.79 | 0.48 | ~ | 0.79 |
| 13200 | 0.66 | 0.29 | ~ | 0.66 |  | 0.75 | 0.47 | ~ | 0.75 |  | 0.86 | 0.52 | ~ | 0.86 |  | 0.91 | 0.50 | ~ | 0.91 |  | 0.89 | 0.48 | ~ | 0.89 |  | 0.80 | 0.49 | ~ | 0.80 |
| 13300 | 0.67 | 0.31 | ~ | 0.67 |  | 0.76 | 0.49 | ~ | 0.76 |  | 0.89 | 0.54 | ~ | 0.89 |  | 0.94 | 0.52 | ~ | 0.94 |  | 0.91 | 0.50 | ~ | 0.91 |  | 0.81 | 0.51 | ~ | 0.81 |
| 13400 | 0.68 | 0.32 | ~ | 0.68 |  | 0.78 | 0.51 | ~ | 0.78 |  | 0.91 | 0.56 | ~ | 0.91 |  | 0.96 | 0.54 | ~ | 0.96 |  | 0.93 | 0.52 | ~ | 0.93 |  | 0.83 | 0.53 | ~ | 0.83 |
| 13500 | 0.69 | 0.34 | ~ | 0.69 |  | 0.80 | 0.53 | ~ | 0.80 |  | 0.94 | 0.58 | ~ | 0.94 |  | 0.99 | 0.56 | ~ | 0.99 |  | 0.95 | 0.54 | ~ | 0.95 |  | 0.84 | 0.55 | ~ | 0.84 |
| 13600 | 0.70 | 0.35 | ~ | 0.70 |  | 0.82 | 0.56 | ~ | 0.82 |  | 0.98 | 0.60 | ~ | 0.98 |  | 1.02 | 0.59 | ~ | 1.02 |  | 0.97 | 0.56 | ~ | 0.97 |  | 0.86 | 0.57 | ~ | 0.86 |
| 13700 | 0.71 | 0.37 | ~ | 0.71 |  | 0.84 | 0.58 | ~ | 0.84 |  | 1.01 | 0.62 | ~ | 1.01 |  | 1.06 | 0.61 | ~ | 1.06 |  | 1.00 | 0.59 | ~ | 1.00 |  | 0.88 | 0.59 | ~ | 0.88 |
| 13800 | 0.73 | 0.39 | ~ | 0.73 |  | 0.87 | 0.60 | ~ | 0.87 |  | 1.05 | 0.64 | ~ | 1.05 |  | 1.09 | 0.64 | ~ | 1.09 |  | 1.02 | 0.62 | ~ | 1.02 |  | 0.90 | 0.61 | ~ | 0.90 |
| 13900 | 0.75 | 0.41 | ~ | 0.75 |  | 0.91 | 0.63 | ~ | 0.91 |  | 1.10 | 0.67 | ~ | 1.10 |  | 1.13 | 0.67 | ~ | 1.13 |  | 1.05 | 0.65 | ~ | 1.05 |  | 0.92 | 0.64 | ~ | 0.92 |
| 14000 | 0.77 | 0.44 | ~ | 0.77 |  | 0.94 | 0.65 | ~ | 0.94 |  | 1.15 | 0.70 | ~ | 1.15 |  | 1.18 | 0.70 | ~ | 1.18 |  | 1.08 | 0.68 | ~ | 1.08 |  | 0.95 | 0.66 | ~ | 0.95 |
| 14100 | 0.79 | 0.46 | ~ | 0.79 |  | 0.99 | 0.67 | ~ | 0.99 |  | 1.20 | 0.72 | ~ | 1.20 |  | 1.22 | 0.73 | ~ | 1.22 |  | 1.12 | 0.71 | ~ | 1.12 |  | 0.98 | 0.69 | ~ | 0.98 |
| 14200 | 0.82 | 0.49 | ~ | 0.82 |  | 1.04 | 0.70 | ~ | 1.04 |  | 1.26 | 0.75 | ~ | 1.26 |  | 1.28 | 0.77 | ~ | 1.28 |  | 1.16 | 0.75 | ~ | 1.16 |  | 1.01 | 0.72 | ~ | 1.01 |
| 14300 | 0.85 | 0.51 | ~ | 0.85 |  | 1.10 | 0.72 | ~ | 1.10 |  | 1.33 | 0.78 | ~ | 1.33 |  | 1.33 | 0.81 | ~ | 1.33 |  | 1.20 | 0.79 | ~ | 1.20 |  | 1.04 | 0.75 | ~ | 1.04 |
| 14400 | 0.89 | 0.54 | ~ | 0.89 |  | 1.17 | 0.75 | ~ | 1.17 |  | 1.41 | 0.81 | ~ | 1.41 |  | 1.39 | 0.85 | ~ | 1.39 |  | 1.25 | 0.84 | ~ | 1.25 |  | 1.09 | 0.78 | ~ | 1.09 |
| 14500 | 0.94 | 0.56 | ~ | 0.94 |  | 1.24 | 0.77 | ~ | 1.24 |  | 1.49 | 0.84 | ~ | 1.49 |  | 1.46 | 0.89 | ~ | 1.46 |  | 1.30 | 0.88 | ~ | 1.30 |  | 1.13 | 0.82 | ~ | 1.13 |
| 14600 | 1.00 | 0.59 | ~ | 1.00 |  | 1.33 | 0.79 | ~ | 1.33 |  | 1.58 | 0.88 | ~ | 1.58 |  | 1.54 | 0.94 | ~ | 1.54 |  | 1.36 | 0.93 | ~ | 1.36 |  | 1.18 | 0.85 | ~ | 1.18 |
| 14700 | 1.06 | 0.62 | ~ | 1.06 |  | 1.43 | 0.82 | ~ | 1.43 |  | 1.69 | 0.91 | ~ | 1.69 |  | 1.62 | 0.99 | ~ | 1.62 |  | 1.43 | 0.98 | ~ | 1.43 |  | 1.25 | 0.89 | ~ | 1.25 |
| 14800 | 1.14 | 0.64 | ~ | 1.14 |  | 1.54 | 0.84 | ~ | 1.54 |  | 1.80 | 0.95 | ~ | 1.80 |  | 1.72 | 1.05 | ~ | 1.72 |  | 1.51 | 1.04 | ~ | 1.51 |  | 1.31 | 0.92 | ~ | 1.31 |
| 14900 | 1.23 | 0.67 | ~ | 1.23 |  | 1.67 | 0.86 | ~ | 1.67 |  | 1.94 | 0.99 | ~ | 1.94 |  | 1.82 | 1.10 | ~ | 1.82 |  | 1.60 | 1.09 | ~ | 1.60 |  | 1.39 | 0.96 | ~ | 1.39 |
| 15000 | 1.34 | 0.69 | ~ | 1.34 |  | 1.81 | 0.89 | ~ | 1.81 |  | 2.08 | 1.03 | ~ | 2.08 |  | 1.94 | 1.17 | ~ | 1.94 |  | 1.70 | 1.15 | ~ | 1.70 |  | 1.48 | 1.00 | ~ | 1.48 |
| 15100 | 1.46 | 0.72 | ~ | 1.46 |  | 1.98 | 0.92 | ~ | 1.98 |  | 2.25 | 1.08 | ~ | 2.25 |  | 2.07 | 1.23 | ~ | 2.07 |  | 1.81 | 1.21 | ~ | 1.81 |  | 1.58 | 1.03 | ~ | 1.58 |
| new confirmed COVID-19 | lag6 | | | |  | lag7 | | | |  | lag8 | | | |  | lag9 | | | |  | lag10 | | | |  |  |  |  |  |
| 0 | 1 | 1 | ~ | 1 |  | 1 | 1 | ~ | 1 |  | 1 | 1 | ~ | 1 |  | 1 | 1 | ~ | 1 |  | 1 | 1 | ~ | 1.00 |  |  |  |  |  |
| 100 | 1.07 | 1.02 | ~ | 1.07 |  | 1.05 | 1.00 | ~ | 1.05 |  | 1.03 | 0.97 | ~ | 1.03 |  | 1.00 | 0.95 | ~ | 1.00 |  | 1.01 | 0.92 | ~ | 1.01 |  |  |  |  |  |
| 200 | 1.10 | 1.04 | ~ | 1.10 |  | 1.07 | 1.01 | ~ | 1.07 |  | 1.04 | 0.97 | ~ | 1.04 |  | 1.00 | 0.95 | ~ | 1.00 |  | 1.03 | 0.93 | ~ | 1.03 |  |  |  |  |  |
| 300 | 1.12 | 1.06 | ~ | 1.12 |  | 1.09 | 1.02 | ~ | 1.09 |  | 1.05 | 0.97 | ~ | 1.05 |  | 1.01 | 0.94 | ~ | 1.01 |  | 1.04 | 0.93 | ~ | 1.04 |  |  |  |  |  |
| 400 | 1.15 | 1.07 | ~ | 1.15 |  | 1.11 | 1.03 | ~ | 1.11 |  | 1.06 | 0.97 | ~ | 1.06 |  | 1.02 | 0.94 | ~ | 1.02 |  | 1.06 | 0.93 | ~ | 1.06 |  |  |  |  |  |
| 500 | 1.18 | 1.09 | ~ | 1.18 |  | 1.13 | 1.04 | ~ | 1.13 |  | 1.07 | 0.96 | ~ | 1.07 |  | 1.02 | 0.93 | ~ | 1.02 |  | 1.08 | 0.93 | ~ | 1.08 |  |  |  |  |  |
| 600 | 1.20 | 1.10 | ~ | 1.20 |  | 1.14 | 1.04 | ~ | 1.14 |  | 1.09 | 0.96 | ~ | 1.09 |  | 1.03 | 0.93 | ~ | 1.03 |  | 1.10 | 0.93 | ~ | 1.10 |  |  |  |  |  |
| 700 | 1.23 | 1.11 | ~ | 1.23 |  | 1.16 | 1.05 | ~ | 1.16 |  | 1.10 | 0.95 | ~ | 1.10 |  | 1.03 | 0.92 | ~ | 1.03 |  | 1.11 | 0.93 | ~ | 1.11 |  |  |  |  |  |
| 800 | 1.26 | 1.13 | ~ | 1.26 |  | 1.18 | 1.05 | ~ | 1.18 |  | 1.10 | 0.95 | ~ | 1.10 |  | 1.04 | 0.92 | ~ | 1.04 |  | 1.13 | 0.92 | ~ | 1.13 |  |  |  |  |  |
| 900 | 1.28 | 1.14 | ~ | 1.28 |  | 1.19 | 1.05 | ~ | 1.19 |  | 1.11 | 0.95 | ~ | 1.11 |  | 1.04 | 0.91 | ~ | 1.04 |  | 1.15 | 0.92 | ~ | 1.15 |  |  |  |  |  |
| 1000 | 1.30 | 1.15 | ~ | 1.30 |  | 1.20 | 1.06 | ~ | 1.20 |  | 1.12 | 0.94 | ~ | 1.12 |  | 1.05 | 0.91 | ~ | 1.05 |  | 1.16 | 0.92 | ~ | 1.16 |  |  |  |  |  |
| 1100 | 1.32 | 1.16 | ~ | 1.32 |  | 1.22 | 1.06 | ~ | 1.22 |  | 1.13 | 0.94 | ~ | 1.13 |  | 1.05 | 0.90 | ~ | 1.05 |  | 1.18 | 0.92 | ~ | 1.18 |  |  |  |  |  |
| 1200 | 1.35 | 1.16 | ~ | 1.35 |  | 1.23 | 1.06 | ~ | 1.23 |  | 1.14 | 0.93 | ~ | 1.14 |  | 1.05 | 0.90 | ~ | 1.05 |  | 1.19 | 0.91 | ~ | 1.19 |  |  |  |  |  |
| 1300 | 1.36 | 1.17 | ~ | 1.36 |  | 1.24 | 1.07 | ~ | 1.24 |  | 1.14 | 0.93 | ~ | 1.14 |  | 1.05 | 0.89 | ~ | 1.05 |  | 1.20 | 0.91 | ~ | 1.20 |  |  |  |  |  |
| 1400 | 1.38 | 1.18 | ~ | 1.38 |  | 1.25 | 1.07 | ~ | 1.25 |  | 1.15 | 0.93 | ~ | 1.15 |  | 1.06 | 0.89 | ~ | 1.06 |  | 1.22 | 0.91 | ~ | 1.22 |  |  |  |  |  |
| 1500 | 1.40 | 1.18 | ~ | 1.40 |  | 1.26 | 1.07 | ~ | 1.26 |  | 1.15 | 0.92 | ~ | 1.15 |  | 1.06 | 0.88 | ~ | 1.06 |  | 1.23 | 0.90 | ~ | 1.23 |  |  |  |  |  |
| 1600 | 1.42 | 1.19 | ~ | 1.42 |  | 1.27 | 1.07 | ~ | 1.27 |  | 1.15 | 0.92 | ~ | 1.15 |  | 1.06 | 0.88 | ~ | 1.06 |  | 1.24 | 0.90 | ~ | 1.24 |  |  |  |  |  |
| 1700 | 1.43 | 1.19 | ~ | 1.43 |  | 1.28 | 1.07 | ~ | 1.28 |  | 1.16 | 0.92 | ~ | 1.16 |  | 1.06 | 0.87 | ~ | 1.06 |  | 1.25 | 0.90 | ~ | 1.25 |  |  |  |  |  |
| 1800 | 1.44 | 1.20 | ~ | 1.44 |  | 1.28 | 1.07 | ~ | 1.28 |  | 1.16 | 0.91 | ~ | 1.16 |  | 1.06 | 0.87 | ~ | 1.06 |  | 1.26 | 0.89 | ~ | 1.26 |  |  |  |  |  |
| 1900 | 1.45 | 1.20 | ~ | 1.45 |  | 1.29 | 1.07 | ~ | 1.29 |  | 1.16 | 0.91 | ~ | 1.16 |  | 1.06 | 0.87 | ~ | 1.06 |  | 1.27 | 0.89 | ~ | 1.27 |  |  |  |  |  |
| 2000 | 1.46 | 1.20 | ~ | 1.46 |  | 1.29 | 1.07 | ~ | 1.29 |  | 1.16 | 0.91 | ~ | 1.16 |  | 1.06 | 0.86 | ~ | 1.06 |  | 1.27 | 0.89 | ~ | 1.27 |  |  |  |  |  |
| 2100 | 1.47 | 1.20 | ~ | 1.47 |  | 1.30 | 1.07 | ~ | 1.30 |  | 1.16 | 0.90 | ~ | 1.16 |  | 1.06 | 0.86 | ~ | 1.06 |  | 1.28 | 0.88 | ~ | 1.28 |  |  |  |  |  |
| 2200 | 1.48 | 1.20 | ~ | 1.48 |  | 1.30 | 1.07 | ~ | 1.30 |  | 1.16 | 0.90 | ~ | 1.16 |  | 1.06 | 0.85 | ~ | 1.06 |  | 1.28 | 0.88 | ~ | 1.28 |  |  |  |  |  |
| 2300 | 1.49 | 1.20 | ~ | 1.49 |  | 1.30 | 1.07 | ~ | 1.30 |  | 1.16 | 0.90 | ~ | 1.16 |  | 1.06 | 0.85 | ~ | 1.06 |  | 1.29 | 0.87 | ~ | 1.29 |  |  |  |  |  |
| 2400 | 1.49 | 1.20 | ~ | 1.49 |  | 1.30 | 1.07 | ~ | 1.30 |  | 1.16 | 0.89 | ~ | 1.16 |  | 1.06 | 0.84 | ~ | 1.06 |  | 1.29 | 0.87 | ~ | 1.29 |  |  |  |  |  |
| 2500 | 1.49 | 1.20 | ~ | 1.49 |  | 1.30 | 1.06 | ~ | 1.30 |  | 1.16 | 0.89 | ~ | 1.16 |  | 1.05 | 0.84 | ~ | 1.05 |  | 1.29 | 0.86 | ~ | 1.29 |  |  |  |  |  |
| 2600 | 1.50 | 1.20 | ~ | 1.50 |  | 1.30 | 1.06 | ~ | 1.30 |  | 1.16 | 0.89 | ~ | 1.16 |  | 1.05 | 0.84 | ~ | 1.05 |  | 1.30 | 0.86 | ~ | 1.30 |  |  |  |  |  |
| 2700 | 1.50 | 1.19 | ~ | 1.50 |  | 1.30 | 1.06 | ~ | 1.30 |  | 1.16 | 0.88 | ~ | 1.16 |  | 1.05 | 0.83 | ~ | 1.05 |  | 1.30 | 0.86 | ~ | 1.30 |  |  |  |  |  |
| 2800 | 1.50 | 1.19 | ~ | 1.50 |  | 1.30 | 1.05 | ~ | 1.30 |  | 1.16 | 0.88 | ~ | 1.16 |  | 1.05 | 0.83 | ~ | 1.05 |  | 1.30 | 0.85 | ~ | 1.30 |  |  |  |  |  |
| 2900 | 1.50 | 1.18 | ~ | 1.50 |  | 1.30 | 1.05 | ~ | 1.30 |  | 1.15 | 0.87 | ~ | 1.15 |  | 1.05 | 0.82 | ~ | 1.05 |  | 1.30 | 0.85 | ~ | 1.30 |  |  |  |  |  |
| 3000 | 1.49 | 1.18 | ~ | 1.49 |  | 1.29 | 1.04 | ~ | 1.29 |  | 1.15 | 0.87 | ~ | 1.15 |  | 1.05 | 0.81 | ~ | 1.05 |  | 1.30 | 0.84 | ~ | 1.30 |  |  |  |  |  |
| 3100 | 1.49 | 1.17 | ~ | 1.49 |  | 1.29 | 1.04 | ~ | 1.29 |  | 1.15 | 0.86 | ~ | 1.15 |  | 1.05 | 0.81 | ~ | 1.05 |  | 1.30 | 0.84 | ~ | 1.30 |  |  |  |  |  |
| 3200 | 1.48 | 1.17 | ~ | 1.48 |  | 1.29 | 1.03 | ~ | 1.29 |  | 1.15 | 0.86 | ~ | 1.15 |  | 1.05 | 0.80 | ~ | 1.05 |  | 1.29 | 0.83 | ~ | 1.29 |  |  |  |  |  |
| 3300 | 1.48 | 1.16 | ~ | 1.48 |  | 1.28 | 1.03 | ~ | 1.28 |  | 1.15 | 0.85 | ~ | 1.15 |  | 1.05 | 0.80 | ~ | 1.05 |  | 1.29 | 0.83 | ~ | 1.29 |  |  |  |  |  |
| 3400 | 1.47 | 1.15 | ~ | 1.47 |  | 1.28 | 1.02 | ~ | 1.28 |  | 1.14 | 0.84 | ~ | 1.14 |  | 1.05 | 0.79 | ~ | 1.05 |  | 1.29 | 0.82 | ~ | 1.29 |  |  |  |  |  |
| 3500 | 1.46 | 1.14 | ~ | 1.46 |  | 1.27 | 1.01 | ~ | 1.27 |  | 1.14 | 0.84 | ~ | 1.14 |  | 1.05 | 0.78 | ~ | 1.05 |  | 1.29 | 0.81 | ~ | 1.29 |  |  |  |  |  |
| 3600 | 1.46 | 1.13 | ~ | 1.46 |  | 1.27 | 1.01 | ~ | 1.27 |  | 1.14 | 0.83 | ~ | 1.14 |  | 1.05 | 0.77 | ~ | 1.05 |  | 1.28 | 0.81 | ~ | 1.28 |  |  |  |  |  |
| 3700 | 1.45 | 1.13 | ~ | 1.45 |  | 1.26 | 1.00 | ~ | 1.26 |  | 1.14 | 0.82 | ~ | 1.14 |  | 1.05 | 0.77 | ~ | 1.05 |  | 1.28 | 0.80 | ~ | 1.28 |  |  |  |  |  |
| 3800 | 1.44 | 1.12 | ~ | 1.44 |  | 1.26 | 0.99 | ~ | 1.26 |  | 1.14 | 0.81 | ~ | 1.14 |  | 1.05 | 0.76 | ~ | 1.05 |  | 1.27 | 0.80 | ~ | 1.27 |  |  |  |  |  |
| 3900 | 1.43 | 1.11 | ~ | 1.43 |  | 1.25 | 0.98 | ~ | 1.25 |  | 1.14 | 0.81 | ~ | 1.14 |  | 1.05 | 0.75 | ~ | 1.05 |  | 1.27 | 0.79 | ~ | 1.27 |  |  |  |  |  |
| 4000 | 1.41 | 1.09 | ~ | 1.41 |  | 1.24 | 0.97 | ~ | 1.24 |  | 1.14 | 0.80 | ~ | 1.14 |  | 1.05 | 0.74 | ~ | 1.05 |  | 1.27 | 0.78 | ~ | 1.27 |  |  |  |  |  |
| 4100 | 1.40 | 1.08 | ~ | 1.40 |  | 1.24 | 0.96 | ~ | 1.24 |  | 1.14 | 0.79 | ~ | 1.14 |  | 1.05 | 0.73 | ~ | 1.05 |  | 1.26 | 0.78 | ~ | 1.26 |  |  |  |  |  |
| 4200 | 1.39 | 1.07 | ~ | 1.39 |  | 1.23 | 0.95 | ~ | 1.23 |  | 1.14 | 0.78 | ~ | 1.14 |  | 1.06 | 0.73 | ~ | 1.06 |  | 1.26 | 0.77 | ~ | 1.26 |  |  |  |  |  |
| 4300 | 1.38 | 1.06 | ~ | 1.38 |  | 1.22 | 0.94 | ~ | 1.22 |  | 1.14 | 0.77 | ~ | 1.14 |  | 1.06 | 0.72 | ~ | 1.06 |  | 1.25 | 0.76 | ~ | 1.25 |  |  |  |  |  |
| 4400 | 1.36 | 1.05 | ~ | 1.36 |  | 1.22 | 0.93 | ~ | 1.22 |  | 1.14 | 0.76 | ~ | 1.14 |  | 1.06 | 0.71 | ~ | 1.06 |  | 1.24 | 0.76 | ~ | 1.24 |  |  |  |  |  |
| 4500 | 1.35 | 1.04 | ~ | 1.35 |  | 1.21 | 0.92 | ~ | 1.21 |  | 1.14 | 0.75 | ~ | 1.14 |  | 1.06 | 0.70 | ~ | 1.06 |  | 1.24 | 0.75 | ~ | 1.24 |  |  |  |  |  |
| 4600 | 1.34 | 1.02 | ~ | 1.34 |  | 1.20 | 0.91 | ~ | 1.20 |  | 1.14 | 0.74 | ~ | 1.14 |  | 1.07 | 0.69 | ~ | 1.07 |  | 1.23 | 0.74 | ~ | 1.23 |  |  |  |  |  |
| 4700 | 1.32 | 1.01 | ~ | 1.32 |  | 1.20 | 0.90 | ~ | 1.20 |  | 1.14 | 0.73 | ~ | 1.14 |  | 1.07 | 0.68 | ~ | 1.07 |  | 1.23 | 0.73 | ~ | 1.23 |  |  |  |  |  |
| 4800 | 1.31 | 1.00 | ~ | 1.31 |  | 1.19 | 0.89 | ~ | 1.19 |  | 1.14 | 0.72 | ~ | 1.14 |  | 1.07 | 0.67 | ~ | 1.07 |  | 1.22 | 0.73 | ~ | 1.22 |  |  |  |  |  |
| 4900 | 1.29 | 0.98 | ~ | 1.29 |  | 1.18 | 0.87 | ~ | 1.18 |  | 1.14 | 0.71 | ~ | 1.14 |  | 1.08 | 0.66 | ~ | 1.08 |  | 1.22 | 0.72 | ~ | 1.22 |  |  |  |  |  |
| 5000 | 1.28 | 0.97 | ~ | 1.28 |  | 1.18 | 0.86 | ~ | 1.18 |  | 1.15 | 0.70 | ~ | 1.15 |  | 1.08 | 0.65 | ~ | 1.08 |  | 1.21 | 0.71 | ~ | 1.21 |  |  |  |  |  |
| 5100 | 1.26 | 0.96 | ~ | 1.26 |  | 1.17 | 0.85 | ~ | 1.17 |  | 1.15 | 0.69 | ~ | 1.15 |  | 1.08 | 0.64 | ~ | 1.08 |  | 1.21 | 0.70 | ~ | 1.21 |  |  |  |  |  |
| 5200 | 1.24 | 0.94 | ~ | 1.24 |  | 1.16 | 0.84 | ~ | 1.16 |  | 1.15 | 0.68 | ~ | 1.15 |  | 1.09 | 0.63 | ~ | 1.09 |  | 1.20 | 0.69 | ~ | 1.20 |  |  |  |  |  |
| 5300 | 1.23 | 0.93 | ~ | 1.23 |  | 1.16 | 0.82 | ~ | 1.16 |  | 1.15 | 0.67 | ~ | 1.15 |  | 1.09 | 0.62 | ~ | 1.09 |  | 1.20 | 0.69 | ~ | 1.20 |  |  |  |  |  |
| 5400 | 1.21 | 0.91 | ~ | 1.21 |  | 1.15 | 0.81 | ~ | 1.15 |  | 1.16 | 0.65 | ~ | 1.16 |  | 1.10 | 0.62 | ~ | 1.10 |  | 1.19 | 0.68 | ~ | 1.19 |  |  |  |  |  |
| 5500 | 1.20 | 0.90 | ~ | 1.20 |  | 1.14 | 0.80 | ~ | 1.14 |  | 1.16 | 0.64 | ~ | 1.16 |  | 1.10 | 0.61 | ~ | 1.10 |  | 1.19 | 0.67 | ~ | 1.19 |  |  |  |  |  |
| 5600 | 1.18 | 0.89 | ~ | 1.18 |  | 1.14 | 0.79 | ~ | 1.14 |  | 1.16 | 0.63 | ~ | 1.16 |  | 1.10 | 0.60 | ~ | 1.10 |  | 1.18 | 0.66 | ~ | 1.18 |  |  |  |  |  |
| 5700 | 1.17 | 0.87 | ~ | 1.17 |  | 1.13 | 0.77 | ~ | 1.13 |  | 1.16 | 0.62 | ~ | 1.16 |  | 1.11 | 0.59 | ~ | 1.11 |  | 1.18 | 0.65 | ~ | 1.18 |  |  |  |  |  |
| 5800 | 1.15 | 0.86 | ~ | 1.15 |  | 1.12 | 0.76 | ~ | 1.12 |  | 1.17 | 0.61 | ~ | 1.17 |  | 1.11 | 0.58 | ~ | 1.11 |  | 1.17 | 0.64 | ~ | 1.17 |  |  |  |  |  |
| 5900 | 1.13 | 0.84 | ~ | 1.13 |  | 1.12 | 0.75 | ~ | 1.12 |  | 1.17 | 0.60 | ~ | 1.17 |  | 1.12 | 0.57 | ~ | 1.12 |  | 1.17 | 0.63 | ~ | 1.17 |  |  |  |  |  |
| 6000 | 1.12 | 0.83 | ~ | 1.12 |  | 1.11 | 0.74 | ~ | 1.11 |  | 1.17 | 0.59 | ~ | 1.17 |  | 1.12 | 0.56 | ~ | 1.12 |  | 1.16 | 0.62 | ~ | 1.16 |  |  |  |  |  |
| 6100 | 1.10 | 0.81 | ~ | 1.10 |  | 1.10 | 0.72 | ~ | 1.10 |  | 1.18 | 0.58 | ~ | 1.18 |  | 1.13 | 0.55 | ~ | 1.13 |  | 1.16 | 0.62 | ~ | 1.16 |  |  |  |  |  |
| 6200 | 1.09 | 0.80 | ~ | 1.09 |  | 1.10 | 0.71 | ~ | 1.10 |  | 1.18 | 0.57 | ~ | 1.18 |  | 1.13 | 0.55 | ~ | 1.13 |  | 1.15 | 0.61 | ~ | 1.15 |  |  |  |  |  |
| 6300 | 1.07 | 0.79 | ~ | 1.07 |  | 1.09 | 0.70 | ~ | 1.09 |  | 1.18 | 0.56 | ~ | 1.18 |  | 1.14 | 0.54 | ~ | 1.14 |  | 1.15 | 0.60 | ~ | 1.15 |  |  |  |  |  |
| 6400 | 1.06 | 0.77 | ~ | 1.06 |  | 1.08 | 0.69 | ~ | 1.08 |  | 1.19 | 0.55 | ~ | 1.19 |  | 1.14 | 0.53 | ~ | 1.14 |  | 1.15 | 0.59 | ~ | 1.15 |  |  |  |  |  |
| 6500 | 1.04 | 0.76 | ~ | 1.04 |  | 1.08 | 0.68 | ~ | 1.08 |  | 1.19 | 0.54 | ~ | 1.19 |  | 1.15 | 0.52 | ~ | 1.15 |  | 1.14 | 0.58 | ~ | 1.14 |  |  |  |  |  |
| 6600 | 1.03 | 0.75 | ~ | 1.03 |  | 1.07 | 0.66 | ~ | 1.07 |  | 1.19 | 0.53 | ~ | 1.19 |  | 1.15 | 0.51 | ~ | 1.15 |  | 1.14 | 0.57 | ~ | 1.14 |  |  |  |  |  |
| 6700 | 1.01 | 0.73 | ~ | 1.01 |  | 1.06 | 0.65 | ~ | 1.06 |  | 1.20 | 0.52 | ~ | 1.20 |  | 1.16 | 0.51 | ~ | 1.16 |  | 1.14 | 0.56 | ~ | 1.14 |  |  |  |  |  |
| 6800 | 1.00 | 0.72 | ~ | 1.00 |  | 1.06 | 0.64 | ~ | 1.06 |  | 1.20 | 0.51 | ~ | 1.20 |  | 1.16 | 0.50 | ~ | 1.16 |  | 1.13 | 0.55 | ~ | 1.13 |  |  |  |  |  |
| 6900 | 0.99 | 0.71 | ~ | 0.99 |  | 1.05 | 0.63 | ~ | 1.05 |  | 1.20 | 0.50 | ~ | 1.20 |  | 1.17 | 0.49 | ~ | 1.17 |  | 1.13 | 0.55 | ~ | 1.13 |  |  |  |  |  |
| 7000 | 0.97 | 0.69 | ~ | 0.97 |  | 1.05 | 0.62 | ~ | 1.05 |  | 1.21 | 0.50 | ~ | 1.21 |  | 1.17 | 0.48 | ~ | 1.17 |  | 1.13 | 0.54 | ~ | 1.13 |  |  |  |  |  |
| 7100 | 0.96 | 0.68 | ~ | 0.96 |  | 1.04 | 0.61 | ~ | 1.04 |  | 1.21 | 0.49 | ~ | 1.21 |  | 1.18 | 0.48 | ~ | 1.18 |  | 1.12 | 0.53 | ~ | 1.12 |  |  |  |  |  |
| 7200 | 0.95 | 0.67 | ~ | 0.95 |  | 1.03 | 0.60 | ~ | 1.03 |  | 1.21 | 0.48 | ~ | 1.21 |  | 1.18 | 0.47 | ~ | 1.18 |  | 1.12 | 0.52 | ~ | 1.12 |  |  |  |  |  |
| 7300 | 0.93 | 0.66 | ~ | 0.93 |  | 1.03 | 0.59 | ~ | 1.03 |  | 1.22 | 0.47 | ~ | 1.22 |  | 1.18 | 0.46 | ~ | 1.18 |  | 1.12 | 0.51 | ~ | 1.12 |  |  |  |  |  |
| 7400 | 0.92 | 0.65 | ~ | 0.92 |  | 1.02 | 0.58 | ~ | 1.02 |  | 1.22 | 0.46 | ~ | 1.22 |  | 1.19 | 0.46 | ~ | 1.19 |  | 1.12 | 0.51 | ~ | 1.12 |  |  |  |  |  |
| 7500 | 0.91 | 0.63 | ~ | 0.91 |  | 1.01 | 0.57 | ~ | 1.01 |  | 1.22 | 0.45 | ~ | 1.22 |  | 1.19 | 0.45 | ~ | 1.19 |  | 1.11 | 0.50 | ~ | 1.11 |  |  |  |  |  |
| 7600 | 0.90 | 0.62 | ~ | 0.90 |  | 1.01 | 0.56 | ~ | 1.01 |  | 1.23 | 0.45 | ~ | 1.23 |  | 1.20 | 0.44 | ~ | 1.20 |  | 1.11 | 0.49 | ~ | 1.11 |  |  |  |  |  |
| 7700 | 0.88 | 0.61 | ~ | 0.88 |  | 1.00 | 0.55 | ~ | 1.00 |  | 1.23 | 0.44 | ~ | 1.23 |  | 1.20 | 0.44 | ~ | 1.20 |  | 1.11 | 0.48 | ~ | 1.11 |  |  |  |  |  |
| 7800 | 0.87 | 0.60 | ~ | 0.87 |  | 1.00 | 0.54 | ~ | 1.00 |  | 1.23 | 0.43 | ~ | 1.23 |  | 1.21 | 0.43 | ~ | 1.21 |  | 1.11 | 0.48 | ~ | 1.11 |  |  |  |  |  |
| 7900 | 0.86 | 0.59 | ~ | 0.86 |  | 0.99 | 0.53 | ~ | 0.99 |  | 1.23 | 0.43 | ~ | 1.23 |  | 1.21 | 0.43 | ~ | 1.21 |  | 1.10 | 0.47 | ~ | 1.10 |  |  |  |  |  |
| 8000 | 0.85 | 0.58 | ~ | 0.85 |  | 0.99 | 0.52 | ~ | 0.99 |  | 1.24 | 0.42 | ~ | 1.24 |  | 1.21 | 0.42 | ~ | 1.21 |  | 1.10 | 0.46 | ~ | 1.10 |  |  |  |  |  |
| 8100 | 0.84 | 0.57 | ~ | 0.84 |  | 0.98 | 0.51 | ~ | 0.98 |  | 1.24 | 0.41 | ~ | 1.24 |  | 1.22 | 0.42 | ~ | 1.22 |  | 1.10 | 0.46 | ~ | 1.10 |  |  |  |  |  |
| 8200 | 0.83 | 0.56 | ~ | 0.83 |  | 0.97 | 0.51 | ~ | 0.97 |  | 1.24 | 0.41 | ~ | 1.24 |  | 1.22 | 0.41 | ~ | 1.22 |  | 1.10 | 0.45 | ~ | 1.10 |  |  |  |  |  |
| 8300 | 0.82 | 0.55 | ~ | 0.82 |  | 0.97 | 0.50 | ~ | 0.97 |  | 1.24 | 0.40 | ~ | 1.24 |  | 1.22 | 0.41 | ~ | 1.22 |  | 1.10 | 0.45 | ~ | 1.10 |  |  |  |  |  |
| 8400 | 0.81 | 0.54 | ~ | 0.81 |  | 0.96 | 0.49 | ~ | 0.96 |  | 1.24 | 0.40 | ~ | 1.24 |  | 1.23 | 0.40 | ~ | 1.23 |  | 1.10 | 0.44 | ~ | 1.10 |  |  |  |  |  |
| 8500 | 0.80 | 0.54 | ~ | 0.80 |  | 0.96 | 0.48 | ~ | 0.96 |  | 1.25 | 0.39 | ~ | 1.25 |  | 1.23 | 0.40 | ~ | 1.23 |  | 1.09 | 0.43 | ~ | 1.09 |  |  |  |  |  |
| 8600 | 0.79 | 0.53 | ~ | 0.79 |  | 0.95 | 0.48 | ~ | 0.95 |  | 1.25 | 0.38 | ~ | 1.25 |  | 1.23 | 0.40 | ~ | 1.23 |  | 1.09 | 0.43 | ~ | 1.09 |  |  |  |  |  |
| 8700 | 0.78 | 0.52 | ~ | 0.78 |  | 0.95 | 0.47 | ~ | 0.95 |  | 1.25 | 0.38 | ~ | 1.25 |  | 1.23 | 0.39 | ~ | 1.23 |  | 1.09 | 0.42 | ~ | 1.09 |  |  |  |  |  |
| 8800 | 0.77 | 0.51 | ~ | 0.77 |  | 0.94 | 0.46 | ~ | 0.94 |  | 1.25 | 0.38 | ~ | 1.25 |  | 1.24 | 0.39 | ~ | 1.24 |  | 1.09 | 0.42 | ~ | 1.09 |  |  |  |  |  |
| 8900 | 0.76 | 0.50 | ~ | 0.76 |  | 0.94 | 0.46 | ~ | 0.94 |  | 1.25 | 0.37 | ~ | 1.25 |  | 1.24 | 0.38 | ~ | 1.24 |  | 1.09 | 0.41 | ~ | 1.09 |  |  |  |  |  |
| 9000 | 0.76 | 0.50 | ~ | 0.76 |  | 0.93 | 0.45 | ~ | 0.93 |  | 1.25 | 0.37 | ~ | 1.25 |  | 1.24 | 0.38 | ~ | 1.24 |  | 1.09 | 0.41 | ~ | 1.09 |  |  |  |  |  |
| 9100 | 0.75 | 0.49 | ~ | 0.75 |  | 0.93 | 0.45 | ~ | 0.93 |  | 1.25 | 0.36 | ~ | 1.25 |  | 1.24 | 0.38 | ~ | 1.24 |  | 1.09 | 0.40 | ~ | 1.09 |  |  |  |  |  |
| 9200 | 0.74 | 0.48 | ~ | 0.74 |  | 0.92 | 0.44 | ~ | 0.92 |  | 1.25 | 0.36 | ~ | 1.25 |  | 1.24 | 0.38 | ~ | 1.24 |  | 1.09 | 0.40 | ~ | 1.09 |  |  |  |  |  |
| 9300 | 0.73 | 0.48 | ~ | 0.73 |  | 0.92 | 0.43 | ~ | 0.92 |  | 1.25 | 0.35 | ~ | 1.25 |  | 1.24 | 0.37 | ~ | 1.24 |  | 1.08 | 0.40 | ~ | 1.08 |  |  |  |  |  |
| 9400 | 0.73 | 0.47 | ~ | 0.73 |  | 0.91 | 0.43 | ~ | 0.91 |  | 1.25 | 0.35 | ~ | 1.25 |  | 1.24 | 0.37 | ~ | 1.24 |  | 1.08 | 0.39 | ~ | 1.08 |  |  |  |  |  |
| 9500 | 0.72 | 0.47 | ~ | 0.72 |  | 0.91 | 0.42 | ~ | 0.91 |  | 1.25 | 0.35 | ~ | 1.25 |  | 1.24 | 0.37 | ~ | 1.24 |  | 1.08 | 0.39 | ~ | 1.08 |  |  |  |  |  |
| 9600 | 0.71 | 0.46 | ~ | 0.71 |  | 0.90 | 0.42 | ~ | 0.90 |  | 1.25 | 0.35 | ~ | 1.25 |  | 1.24 | 0.37 | ~ | 1.24 |  | 1.08 | 0.38 | ~ | 1.08 |  |  |  |  |  |
| 9700 | 0.71 | 0.46 | ~ | 0.71 |  | 0.90 | 0.42 | ~ | 0.90 |  | 1.25 | 0.34 | ~ | 1.25 |  | 1.24 | 0.36 | ~ | 1.24 |  | 1.08 | 0.38 | ~ | 1.08 |  |  |  |  |  |
| 9800 | 0.70 | 0.45 | ~ | 0.70 |  | 0.89 | 0.41 | ~ | 0.89 |  | 1.25 | 0.34 | ~ | 1.25 |  | 1.24 | 0.36 | ~ | 1.24 |  | 1.08 | 0.38 | ~ | 1.08 |  |  |  |  |  |
| 9900 | 0.70 | 0.45 | ~ | 0.70 |  | 0.89 | 0.41 | ~ | 0.89 |  | 1.24 | 0.34 | ~ | 1.24 |  | 1.24 | 0.36 | ~ | 1.24 |  | 1.08 | 0.38 | ~ | 1.08 |  |  |  |  |  |
| 10000 | 0.69 | 0.44 | ~ | 0.69 |  | 0.89 | 0.41 | ~ | 0.89 |  | 1.24 | 0.34 | ~ | 1.24 |  | 1.24 | 0.36 | ~ | 1.24 |  | 1.08 | 0.37 | ~ | 1.08 |  |  |  |  |  |
| 10100 | 0.69 | 0.44 | ~ | 0.69 |  | 0.88 | 0.40 | ~ | 0.88 |  | 1.24 | 0.33 | ~ | 1.24 |  | 1.24 | 0.36 | ~ | 1.24 |  | 1.08 | 0.37 | ~ | 1.08 |  |  |  |  |  |
| 10200 | 0.68 | 0.44 | ~ | 0.68 |  | 0.88 | 0.40 | ~ | 0.88 |  | 1.24 | 0.33 | ~ | 1.24 |  | 1.24 | 0.36 | ~ | 1.24 |  | 1.08 | 0.37 | ~ | 1.08 |  |  |  |  |  |
| 10300 | 0.68 | 0.43 | ~ | 0.68 |  | 0.87 | 0.40 | ~ | 0.87 |  | 1.23 | 0.33 | ~ | 1.23 |  | 1.23 | 0.36 | ~ | 1.23 |  | 1.08 | 0.37 | ~ | 1.08 |  |  |  |  |  |
| 10400 | 0.67 | 0.43 | ~ | 0.67 |  | 0.87 | 0.40 | ~ | 0.87 |  | 1.23 | 0.33 | ~ | 1.23 |  | 1.23 | 0.36 | ~ | 1.23 |  | 1.08 | 0.37 | ~ | 1.08 |  |  |  |  |  |
| 10500 | 0.67 | 0.43 | ~ | 0.67 |  | 0.87 | 0.39 | ~ | 0.87 |  | 1.23 | 0.33 | ~ | 1.23 |  | 1.23 | 0.36 | ~ | 1.23 |  | 1.08 | 0.37 | ~ | 1.08 |  |  |  |  |  |
| 10600 | 0.67 | 0.43 | ~ | 0.67 |  | 0.86 | 0.39 | ~ | 0.86 |  | 1.22 | 0.33 | ~ | 1.22 |  | 1.22 | 0.36 | ~ | 1.22 |  | 1.08 | 0.36 | ~ | 1.08 |  |  |  |  |  |
| 10700 | 0.66 | 0.43 | ~ | 0.66 |  | 0.86 | 0.39 | ~ | 0.86 |  | 1.22 | 0.33 | ~ | 1.22 |  | 1.22 | 0.36 | ~ | 1.22 |  | 1.08 | 0.36 | ~ | 1.08 |  |  |  |  |  |
| 10800 | 0.66 | 0.42 | ~ | 0.66 |  | 0.86 | 0.39 | ~ | 0.86 |  | 1.21 | 0.33 | ~ | 1.21 |  | 1.21 | 0.36 | ~ | 1.21 |  | 1.08 | 0.36 | ~ | 1.08 |  |  |  |  |  |
| 10900 | 0.66 | 0.42 | ~ | 0.66 |  | 0.85 | 0.39 | ~ | 0.85 |  | 1.21 | 0.33 | ~ | 1.21 |  | 1.21 | 0.36 | ~ | 1.21 |  | 1.08 | 0.36 | ~ | 1.08 |  |  |  |  |  |
| 11000 | 0.66 | 0.42 | ~ | 0.66 |  | 0.85 | 0.39 | ~ | 0.85 |  | 1.20 | 0.33 | ~ | 1.20 |  | 1.20 | 0.36 | ~ | 1.20 |  | 1.08 | 0.36 | ~ | 1.08 |  |  |  |  |  |
| 11100 | 0.65 | 0.42 | ~ | 0.65 |  | 0.85 | 0.39 | ~ | 0.85 |  | 1.20 | 0.33 | ~ | 1.20 |  | 1.20 | 0.36 | ~ | 1.20 |  | 1.08 | 0.36 | ~ | 1.08 |  |  |  |  |  |
| 11200 | 0.65 | 0.42 | ~ | 0.65 |  | 0.85 | 0.39 | ~ | 0.85 |  | 1.19 | 0.33 | ~ | 1.19 |  | 1.19 | 0.37 | ~ | 1.19 |  | 1.08 | 0.36 | ~ | 1.08 |  |  |  |  |  |
| 11300 | 0.65 | 0.42 | ~ | 0.65 |  | 0.84 | 0.39 | ~ | 0.84 |  | 1.18 | 0.33 | ~ | 1.18 |  | 1.19 | 0.37 | ~ | 1.19 |  | 1.08 | 0.37 | ~ | 1.08 |  |  |  |  |  |
| 11400 | 0.65 | 0.42 | ~ | 0.65 |  | 0.84 | 0.39 | ~ | 0.84 |  | 1.18 | 0.33 | ~ | 1.18 |  | 1.18 | 0.37 | ~ | 1.18 |  | 1.08 | 0.37 | ~ | 1.08 |  |  |  |  |  |
| 11500 | 0.65 | 0.43 | ~ | 0.65 |  | 0.84 | 0.39 | ~ | 0.84 |  | 1.17 | 0.34 | ~ | 1.17 |  | 1.17 | 0.37 | ~ | 1.17 |  | 1.08 | 0.37 | ~ | 1.08 |  |  |  |  |  |
| 11600 | 0.65 | 0.43 | ~ | 0.65 |  | 0.84 | 0.39 | ~ | 0.84 |  | 1.16 | 0.34 | ~ | 1.16 |  | 1.16 | 0.38 | ~ | 1.16 |  | 1.08 | 0.37 | ~ | 1.08 |  |  |  |  |  |
| 11700 | 0.65 | 0.43 | ~ | 0.65 |  | 0.84 | 0.39 | ~ | 0.84 |  | 1.15 | 0.34 | ~ | 1.15 |  | 1.15 | 0.38 | ~ | 1.15 |  | 1.08 | 0.37 | ~ | 1.08 |  |  |  |  |  |
| 11800 | 0.65 | 0.43 | ~ | 0.65 |  | 0.83 | 0.40 | ~ | 0.83 |  | 1.15 | 0.34 | ~ | 1.15 |  | 1.15 | 0.38 | ~ | 1.15 |  | 1.08 | 0.37 | ~ | 1.08 |  |  |  |  |  |
| 11900 | 0.66 | 0.44 | ~ | 0.66 |  | 0.83 | 0.40 | ~ | 0.83 |  | 1.14 | 0.35 | ~ | 1.14 |  | 1.14 | 0.39 | ~ | 1.14 |  | 1.09 | 0.38 | ~ | 1.09 |  |  |  |  |  |
| 12000 | 0.66 | 0.44 | ~ | 0.66 |  | 0.83 | 0.40 | ~ | 0.83 |  | 1.13 | 0.35 | ~ | 1.13 |  | 1.13 | 0.39 | ~ | 1.13 |  | 1.09 | 0.38 | ~ | 1.09 |  |  |  |  |  |
| 12100 | 0.66 | 0.44 | ~ | 0.66 |  | 0.83 | 0.40 | ~ | 0.83 |  | 1.12 | 0.35 | ~ | 1.12 |  | 1.12 | 0.40 | ~ | 1.12 |  | 1.09 | 0.38 | ~ | 1.09 |  |  |  |  |  |
| 12200 | 0.66 | 0.45 | ~ | 0.66 |  | 0.83 | 0.41 | ~ | 0.83 |  | 1.11 | 0.36 | ~ | 1.11 |  | 1.11 | 0.40 | ~ | 1.11 |  | 1.09 | 0.39 | ~ | 1.09 |  |  |  |  |  |
| 12300 | 0.67 | 0.45 | ~ | 0.67 |  | 0.83 | 0.41 | ~ | 0.83 |  | 1.10 | 0.36 | ~ | 1.10 |  | 1.10 | 0.41 | ~ | 1.10 |  | 1.09 | 0.39 | ~ | 1.09 |  |  |  |  |  |
| 12400 | 0.67 | 0.46 | ~ | 0.67 |  | 0.83 | 0.42 | ~ | 0.83 |  | 1.09 | 0.37 | ~ | 1.09 |  | 1.09 | 0.42 | ~ | 1.09 |  | 1.09 | 0.40 | ~ | 1.09 |  |  |  |  |  |
| 12500 | 0.68 | 0.46 | ~ | 0.68 |  | 0.83 | 0.42 | ~ | 0.83 |  | 1.08 | 0.38 | ~ | 1.08 |  | 1.08 | 0.42 | ~ | 1.08 |  | 1.10 | 0.40 | ~ | 1.10 |  |  |  |  |  |
| 12600 | 0.68 | 0.47 | ~ | 0.68 |  | 0.83 | 0.43 | ~ | 0.83 |  | 1.07 | 0.38 | ~ | 1.07 |  | 1.07 | 0.43 | ~ | 1.07 |  | 1.10 | 0.41 | ~ | 1.10 |  |  |  |  |  |
| 12700 | 0.69 | 0.48 | ~ | 0.69 |  | 0.83 | 0.43 | ~ | 0.83 |  | 1.06 | 0.39 | ~ | 1.06 |  | 1.05 | 0.44 | ~ | 1.05 |  | 1.10 | 0.41 | ~ | 1.10 |  |  |  |  |  |
| 12800 | 0.69 | 0.49 | ~ | 0.69 |  | 0.83 | 0.44 | ~ | 0.83 |  | 1.05 | 0.40 | ~ | 1.05 |  | 1.04 | 0.45 | ~ | 1.04 |  | 1.10 | 0.42 | ~ | 1.10 |  |  |  |  |  |
| 12900 | 0.70 | 0.49 | ~ | 0.70 |  | 0.83 | 0.45 | ~ | 0.83 |  | 1.04 | 0.40 | ~ | 1.04 |  | 1.03 | 0.45 | ~ | 1.03 |  | 1.11 | 0.43 | ~ | 1.11 |  |  |  |  |  |
| 13000 | 0.71 | 0.50 | ~ | 0.71 |  | 0.83 | 0.46 | ~ | 0.83 |  | 1.03 | 0.41 | ~ | 1.03 |  | 1.02 | 0.46 | ~ | 1.02 |  | 1.11 | 0.44 | ~ | 1.11 |  |  |  |  |  |
| 13100 | 0.72 | 0.51 | ~ | 0.72 |  | 0.83 | 0.46 | ~ | 0.83 |  | 1.01 | 0.42 | ~ | 1.01 |  | 1.01 | 0.47 | ~ | 1.01 |  | 1.11 | 0.44 | ~ | 1.11 |  |  |  |  |  |
| 13200 | 0.73 | 0.52 | ~ | 0.73 |  | 0.83 | 0.47 | ~ | 0.83 |  | 1.00 | 0.43 | ~ | 1.00 |  | 1.00 | 0.48 | ~ | 1.00 |  | 1.12 | 0.45 | ~ | 1.12 |  |  |  |  |  |
| 13300 | 0.74 | 0.53 | ~ | 0.74 |  | 0.84 | 0.48 | ~ | 0.84 |  | 0.99 | 0.44 | ~ | 0.99 |  | 0.98 | 0.50 | ~ | 0.98 |  | 1.12 | 0.46 | ~ | 1.12 |  |  |  |  |  |
| 13400 | 0.75 | 0.55 | ~ | 0.75 |  | 0.84 | 0.49 | ~ | 0.84 |  | 0.98 | 0.45 | ~ | 0.98 |  | 0.97 | 0.51 | ~ | 0.97 |  | 1.13 | 0.47 | ~ | 1.13 |  |  |  |  |  |
| 13500 | 0.76 | 0.56 | ~ | 0.76 |  | 0.84 | 0.50 | ~ | 0.84 |  | 0.97 | 0.46 | ~ | 0.97 |  | 0.96 | 0.52 | ~ | 0.96 |  | 1.13 | 0.48 | ~ | 1.13 |  |  |  |  |  |
| 13600 | 0.78 | 0.57 | ~ | 0.78 |  | 0.85 | 0.51 | ~ | 0.85 |  | 0.96 | 0.48 | ~ | 0.96 |  | 0.95 | 0.53 | ~ | 0.95 |  | 1.14 | 0.49 | ~ | 1.14 |  |  |  |  |  |
| 13700 | 0.79 | 0.59 | ~ | 0.79 |  | 0.85 | 0.53 | ~ | 0.85 |  | 0.94 | 0.49 | ~ | 0.94 |  | 0.94 | 0.55 | ~ | 0.94 |  | 1.15 | 0.51 | ~ | 1.15 |  |  |  |  |  |
| 13800 | 0.81 | 0.60 | ~ | 0.81 |  | 0.85 | 0.54 | ~ | 0.85 |  | 0.93 | 0.50 | ~ | 0.93 |  | 0.93 | 0.56 | ~ | 0.93 |  | 1.16 | 0.52 | ~ | 1.16 |  |  |  |  |  |
| 13900 | 0.83 | 0.62 | ~ | 0.83 |  | 0.86 | 0.55 | ~ | 0.86 |  | 0.92 | 0.52 | ~ | 0.92 |  | 0.92 | 0.57 | ~ | 0.92 |  | 1.17 | 0.53 | ~ | 1.17 |  |  |  |  |  |
| 14000 | 0.85 | 0.63 | ~ | 0.85 |  | 0.87 | 0.57 | ~ | 0.87 |  | 0.91 | 0.53 | ~ | 0.91 |  | 0.91 | 0.58 | ~ | 0.91 |  | 1.18 | 0.54 | ~ | 1.18 |  |  |  |  |  |
| 14100 | 0.88 | 0.65 | ~ | 0.88 |  | 0.87 | 0.58 | ~ | 0.87 |  | 0.90 | 0.55 | ~ | 0.90 |  | 0.91 | 0.60 | ~ | 0.91 |  | 1.19 | 0.56 | ~ | 1.19 |  |  |  |  |  |
| 14200 | 0.90 | 0.67 | ~ | 0.90 |  | 0.88 | 0.60 | ~ | 0.88 |  | 0.90 | 0.57 | ~ | 0.90 |  | 0.91 | 0.61 | ~ | 0.91 |  | 1.20 | 0.57 | ~ | 1.20 |  |  |  |  |  |
| 14300 | 0.93 | 0.69 | ~ | 0.93 |  | 0.89 | 0.62 | ~ | 0.89 |  | 0.89 | 0.58 | ~ | 0.89 |  | 0.91 | 0.62 | ~ | 0.91 |  | 1.22 | 0.59 | ~ | 1.22 |  |  |  |  |  |
| 14400 | 0.96 | 0.71 | ~ | 0.96 |  | 0.90 | 0.63 | ~ | 0.90 |  | 0.89 | 0.60 | ~ | 0.89 |  | 0.91 | 0.62 | ~ | 0.91 |  | 1.23 | 0.60 | ~ | 1.23 |  |  |  |  |  |
| 14500 | 0.99 | 0.73 | ~ | 0.99 |  | 0.91 | 0.65 | ~ | 0.91 |  | 0.89 | 0.61 | ~ | 0.89 |  | 0.92 | 0.63 | ~ | 0.92 |  | 1.25 | 0.62 | ~ | 1.25 |  |  |  |  |  |
| 14600 | 1.03 | 0.75 | ~ | 1.03 |  | 0.93 | 0.67 | ~ | 0.93 |  | 0.90 | 0.62 | ~ | 0.90 |  | 0.94 | 0.63 | ~ | 0.94 |  | 1.27 | 0.63 | ~ | 1.27 |  |  |  |  |  |
| 14700 | 1.07 | 0.78 | ~ | 1.07 |  | 0.95 | 0.69 | ~ | 0.95 |  | 0.91 | 0.63 | ~ | 0.91 |  | 0.96 | 0.63 | ~ | 0.96 |  | 1.30 | 0.65 | ~ | 1.30 |  |  |  |  |  |
| 14800 | 1.12 | 0.80 | ~ | 1.12 |  | 0.97 | 0.70 | ~ | 0.97 |  | 0.93 | 0.63 | ~ | 0.93 |  | 0.99 | 0.62 | ~ | 0.99 |  | 1.33 | 0.66 | ~ | 1.33 |  |  |  |  |  |
| 14900 | 1.17 | 0.82 | ~ | 1.17 |  | 1.00 | 0.72 | ~ | 1.00 |  | 0.95 | 0.63 | ~ | 0.95 |  | 1.02 | 0.61 | ~ | 1.02 |  | 1.36 | 0.68 | ~ | 1.36 |  |  |  |  |  |
| 15000 | 1.23 | 0.85 | ~ | 1.23 |  | 1.02 | 0.73 | ~ | 1.02 |  | 0.98 | 0.63 | ~ | 0.98 |  | 1.06 | 0.60 | ~ | 1.06 |  | 1.40 | 0.69 | ~ | 1.40 |  |  |  |  |  |
| 15100 | 1.29 | 0.88 | ~ | 1.29 |  | 1.06 | 0.75 | ~ | 1.06 |  | 1.02 | 0.62 | ~ | 1.02 |  | 1.10 | 0.59 | ~ | 1.10 |  | 1.44 | 0.71 | ~ | 1.44 |  |  |  |  |  |
|  |  |  |  |  |  |  |  |  |  |  |  |  |  |  |  |  |  |  |  |  |  |  |  |  |  |  |  |  |  |
